# Signal Perceptron: On the Identifiability of Boolean Function Spaces and Beyond

**DOI:** 10.3389/frai.2022.770254

**Published:** 2022-06-02

**Authors:** Miguel-Angel Mendez Lucero, Rafael-Michael Karampatsis, Enrique Bojorquez Gallardo, Vaishak Belle

**Affiliations:** ^1^The University of Edinburgh, Edinburgh, United Kingdom; ^2^Alan Turing Institute, London, United Kingdom

**Keywords:** signal perceptron, perceptron, learning function spaces, parallel machines, neural networks

## Abstract

In a seminal book, Minsky and Papert define the perceptron as a limited implementation of what they called “parallel machines.” They showed that some binary Boolean functions including XOR are not definable in a single layer perceptron due to its limited capacity to learn only linearly separable functions. In this work, we propose a new more powerful implementation of such parallel machines. This new mathematical tool is defined using analytic sinusoids—instead of linear combinations—to form an analytic signal representation of the function that we want to learn. We show that this re-formulated parallel mechanism can learn, with a single layer, any non-linear *k*-ary Boolean function. Finally, to provide an example of its practical applications, we show that it outperforms the single hidden layer multilayer perceptron in both Boolean function learning and image classification tasks, while also being faster and requiring fewer parameters.

## 1. Introduction

During the last decade, machine learning has pushed the boundaries of what is possible in most, if not every, subdomain of artificial intelligence where training data is available. Examples include but are not limited to natural language processing (Devlin et al., 2019), image classification (Simonyan and Zisserman, 2015; He et al., 2016), cyber security (Ferrag et al., 2020), and reinforcement learning (Silver et al., 2016). Most neural networks architectures such as feed-forward neural networks (also called multilayer perceptrons) (Bebis and Georgiopoulos, 1994) contain perceptrons as one of their fundamental elements. However, as a function approximator, this learning mechanism poses a major limitation, it can only learn linearly separable patterns (Minsky, 1969). It is shown that, for the binary Boolean function space, only 14 of the 16 possible functions are linearly separable. Additionally,when increasing the amount of variables, the number of linearly separable functions decreases exponentially (Gruzling, 2007). Therefore, to approximate any function from a Boolean function space, a multilayer perceptron (MLP) with at least one hidden layer is required (Baum, 1988; Cybenko, 1989). These proofs opened up a whole branch of analysis of theoretical bounds for MLP architectures (Stathakis, 2009). Huang and Babri (1997) specifically showed that the single hidden layer MLP with sigmoid activation function requires that the number of hidden nodes must be as high as the number of training samples in order to learn a small error function approximation. This can be problematic, as shown in later since the number of training samples needed to approximate any function from a function space can increase exponentially. Furthermore, such theoretical analysis only discusses a particular architecture, this can also be problematic as each new architecture requires an in-depth analysis. That is, the problem of finding topological bounds becomes intractable as the amount of layers and activation functions changes. The same problem arises with novel types of perceptrons, such as complex-valued neurons, generalized neurons, morphological neurons, and wavelet neurons (Kulkarni and Venayagamoorthy, 2009; Zhang, 2013; Mondal et al., 2020), which can learn **but not all** non-linear separable functions with a single layer.

We believe that overcoming the issue of non-linear separability for a single mathematical structure is a step forward toward processing optimization and easier analysis for more complex machine learning models such as the ones previously mentioned. In order to solve this problem, we appeal to the mathematical definition of function learning mechanisms, namely parallel machines (Minsky, 1969), that can learn a subset of functions from a given function space. In this work, we are specifically concerned with the type of parallel machines which are able to learn any function from finite function spaces.

Consequently, the main purpose of this paper is to provide a new parallel machine that combines signal processing and neural network techniques in order to create a new kind of function learning mechanism. This new type of mechanism will extend the capabilities of current single layer perceptrons for learning any function from a function space where the domain and co-domain are finite^1^. Ideally, we would like such a mechanism to be able to define any function space with as few parameters as possible. To address this issue, we focus on mechanisms that use linear combination of analytic signals instead of affine functions.

We will show that this mechanism is able to learn whole Boolean function spaces. For Boolean functions, a multilayer perceptron (MLP) needs at least one hidden layer to approximate any function from the *k*-ary Boolean function space with almost negligible error (Hertz et al., 1991). The same follows for more novel architectures where multiple layers are required. Our proposed mechanism can learn any function with just a single layer and without error. Furthermore, unlike its MLP counterpart, its definition doesn't require to specify a particular activation function and requires less operations to compute the function. This result in a more efficient learning method with smaller spatial and computational complexity as we will show later.

Our contributions can be summarized as follows: First, we discuss how the ideas of analytic signals and neural networks can be interrelated by providing the necessary background theory. Second, we introduce a new function learning mechanism based on a linear combination of analytic signals, the **signal perceptron**, along with the necessary mathematical definitions. Third, we provide a definition of the Rosenblatt perceptron (Rosenblatt, 1958) and the MLP with one hidden layer in terms of parallel machines, in order to demonstrate their structural differences against the signal perceptron. Fourth, we provide the mathematical proof that demonstrates the capability of the signal perceptron to learn any function from any *k*-ary Boolean function space, avoiding a topological analysis of the architecture which is required for its counterparts (unless an equivalent proof is devised). Fifth, we showcase that our novel mechanism can be trained using two different learning method algorithms along with their corresponding pseudocode. Sixth, we further propose two more variations of the signal perceptron defined over the real space, in order to use the signal perceptron to solve practical problems such as image classification. We compare such variations along with the signal perceptron and demonstrate their experimental advantages over the MLP, including some practical computer vision problems. Finally, we provide a discussion where we summarize the main advantages of the signal perceptron against other neurons from the literature that attempt to solve the non-linear separability problem, followed by the potential limitations of the signal perceptron and promising directions for future work.

## 2. Related Work

The perceptron has been an essential component of function approximation methods, and its inherent issues have resulted in several important lines of work (Kim and Adali, 2002; Zhang, 2013; Tavanaei et al., 2019; Mondal et al., 2020). These attempts extend or modify the original perceptron so that it can operate over different domains, highlighting the importance of extending the original idea. Unfortunately, these extensions do not solve the problem of learning the linear separable functions with a single layer, they must necessarily have multiple layers. Extensions that try to solve this issue with a single layer are usually defined over bigger domains, for instance, many studies focus on extensions that operate over complex values (Clarke, 1990; Cheolwoo and Daesik, 1998; Kim and Adali, 2002). Such extensions also provide limited solutions to the linear separability problem. As an example, Nitta (2003) encodes real inputs into complex value scalars and defines a linear combination of complex values and weights. This transforms the perceptron into a mapping ℝ → ℂ → ℝ. Since Boolean functions are mappings of the form 𝔹 → 𝔹 rigorous rules and specific activation functions are required to perform such encoding and decoding. Moreover, the linear separability analysis is performed only over the binary Boolean function space (Boolean functions of only two variables). As an extension of that, Amin et al. (2008) includes an experimental analysis that attempts to compute every ternary Boolean function. However, it fails to learn every function thus proving that this mechanism can only learn non-linear functions of arity 2 or less.

The perceptron's importance as a mathematical structure is not limited to computer science, several areas have proposed their versions to overcome its issues or exploit certain characteristics in their domains. Some examples of these new types of perceptrons are: software perceptrons (Rosenblatt, 1958; Maass, 1997; Ritter and Urcid, 2003; Huh and Sejnowski, 2018), biochemical perceptrons (Cazé et al., 2013; Banda and Teuscher, 2014; Blount et al., 2017), and hardware perceptrons (Pisarev et al., 2020). In Section 6.2 we will provide a comparative analysis against complex-valued neurons and other prominent proposals of neurons against our proposed architecture to remark the contributions of this paper.

It is also important to mention the theoretical analysis of the MLP which was the proposed solution for solving the non-linear separability problem (Baum, 1988; Cybenko, 1989). As stated in Section 1, some effort toward defining the topological boundaries for MLPs (Kůrková, 1992; Huang and Babri, 1997; Huang, 2003) has been conducted, but it has been limited to particular architectures and particular activation functions. Furthermore, the analysis over the amount of parameters is assessed by the amount of training samples (i.e., sample complexity). Since we are interested in mechanisms that are able to learn the whole functional space, analysis for the topological bounds should consider the scenario when the training samples equals the size of the domain. An example of this type of analysis was done by Baum (1988), which proved that the single hidden layer MLP can learn any formula in disjunctive normal form. We will show that thanks to this analysis, in conjunction with Huang and Babri (1997), we can calculate the correct spatial complexity of a single hidden layer MLP that can learn any *k*-ary Boolean function.

## 3. Background Theory Description

### 3.1. Function Spaces

Most of the discussion in this paper will be directed toward mathematical structures that are able to learn functions from a finite function space. The finite function space Δ is defined as the set of all possible functions that have domain ***m***^***k***^, where ***m***^***k***^ is the set {(*x*^0^, ..., *x*^*k*−1^) | *x*^*j*^ ∈ *m*}, and co-domain *n*. When a function-learning structure is defined, we expect that such a mathematical method will at least be able to learn a subset of the function space. The perceptron defined by Rosenblatt (Rosenblatt, 1958) is a clear example of such a method, where the subset of learnable functions are the linearly separable functions. By extension, the closer a method is to learning the entirety of a function space, the more expressive will the structure be. Due to this, in order to properly assess how expressive a given method is, it is important to first understand how to calculate a function space's size.

The function space size |Δ| is the number of all possible functions from a given function space Δ and is defined by the formula:


(1) $$\begin{array}{l} |\Delta |=n^{m^{k}} \end{array}$$

where *k* is the amount of variables taken by the function (i.e., the arity of the function), while *n* and *m*^*k*^ are the number of elements that belong to the co-domain *n* and domain ***m***^***k***^, respectively.

For simplicity, this study focuses on the type of function spaces where the set of elements of the domain and co-domain are identical. That is, function spaces of the form {0, 1...., *n* − 1}^*k*^ → {0, 1...., *n* − 1}. However, most of our analysis will revolve around the two element function space (i.e., the *k*-ary Boolean function space).

Shannon (1938) showed that it is possible to obtain any *k*-ary Boolean function from multiple simple (*k* − 1)-ary functions. This achievement demonstrated that any Boolean function can be obtained by its corresponding circuit. Furthermore, Shannon recalls that in infinitesimal calculus it is shown that any function (providing it is continuous and all derivatives are continuous) may be expanded in a Taylor Series. By analogy, proving that the new learning mechanism can also learn any function from the *k*-ary Boolean function space would be a step toward proving that it is also able to express any function. To this end, we next provide the formal definition of a *k*-ary Boolean function space.

**Definition 1**. The k-ary Boolean function space is a set that contains all possible k-ary Boolean functions. A Boolean k-ary function is a mapping of the form {0, 1}^*k*^ → {0, 1}. Where {0, 1} is the Boolean set 𝔹 and *k* is a natural number that defines the number of variables used.

Based on Definition 1 and Equation 1, the size of a k-ary Boolean function space is given by the formula 2^2^*k*^^. Thus, the amount of possible functions for the unary and binary Boolean function spaces will be 4 and 16, respectively. These are shown in Tables 1, 2.

**Table 1:** Every possible unary Boolean function.

***x*_**1**_**	***f*_**0**_**	***f*_**1**_**	***f*_**2**_**	***f*_**3**_**
0	0	1	0	1
1	0	0	1	1

**Table 2:** Every possible binary Boolean function (a.k.a logical connectives).

**(*x*_2_, *x*_1_)**	***f*_**0**_**	***f*_**1**_**	***f*_**2**_**	***f*_**3**_**	***f*_**4**_**	***f*_**5**_**	***f*_**6**_**	***f*_**7**_**	***f*_**8**_**	***f*_**9**_**	***f*_**10**_**	***f*_**11**_**	***f*_**12**_**	***f*_**13**_**	***f*_**14**_**	***f*_**15**_**
(0, 0)	0	0	0	0	0	0	0	0	1	1	1	1	1	1	1	1
(0, 1)	0	0	0	0	1	1	1	1	0	0	0	0	1	1	1	1
(1, 0)	0	0	1	1	0	0	1	1	0	0	1	1	0	0	1	1
(1, 1)	0	1	0	1	0	1	0	1	0	1	0	1	0	1	0	1

Table 1 contains the trivial functions: *f*_0_ (*False*), *f*_1_ (*inverse function*), *f*_2_ (*identity function*) and *f*_3_ (*True*). The same follows in Table 2, which showcases the logic connectives for all binary Boolean functions. We can find some of the most common functions used in propositional logic which are *f*_1_ *(AND* *operator)*, *f*_7_ *(OR* *operator)*, and *f*_6_ *(XOR* *operator)*.

### 3.2. Parallel Machines and the Rosenblatt Perceptron

**Figure 1:**
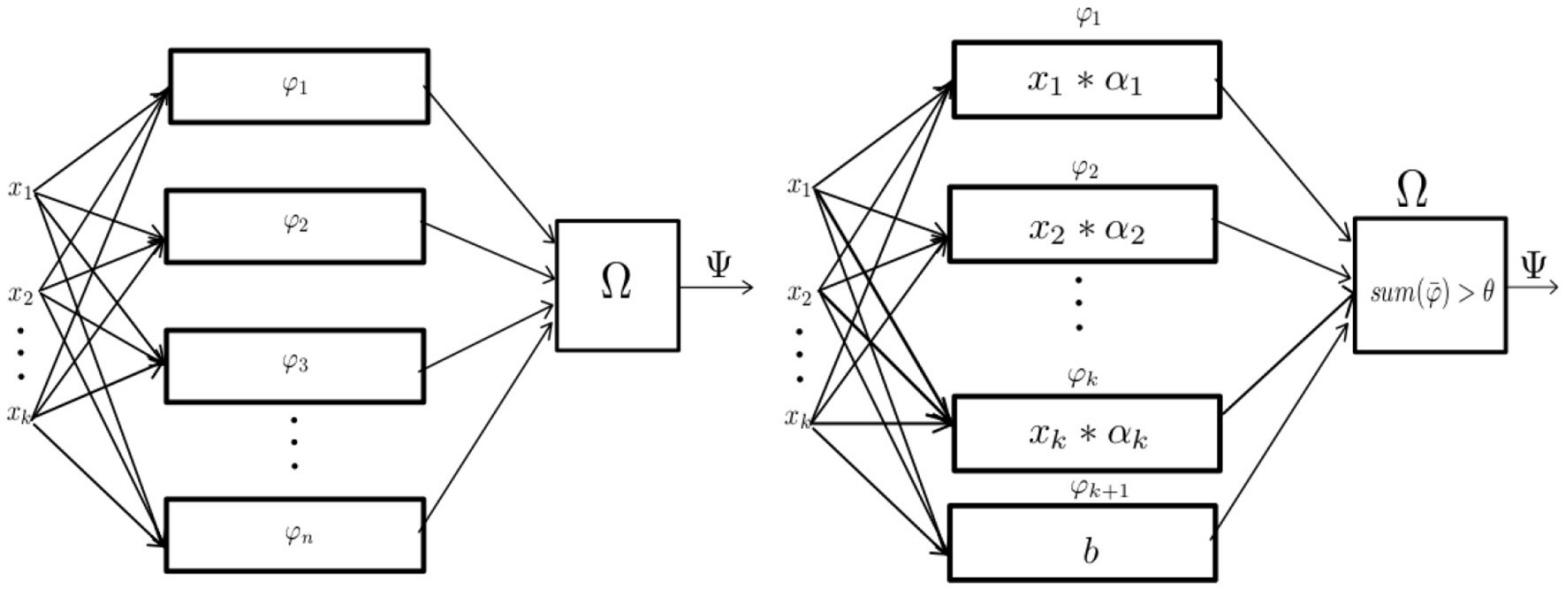
From left to right: The abstract definition of parallel machine and the perceptron as a parametric implementation of the parallel machine. Figures based on the diagrams depicted in the book *Perceptrons* (Minsky, 1969).

The perceptron or Rosenblatt perceptron, is defined as a mathematical method used for binary classification (Rosenblatt, 1958). Such a mechanism is described as a particular implementation of a mathematical structure called a “parallel machine” (as shown in Figure 1). Parallel machines are mathematical structures that make decisions by adding evidence obtained from many small experiments (Minsky, 1969). This is gathered by calculating a set of partial operations that can be computed simultaneously, thus the name “parallel machine.”

Following the ideas of Section 3.1 we will treat parallel machines as mathematical structures that learn a subset of functions from some function space of the form {0, ..., *n* − 1}^*k*^ → {0, ..., *n* − 1}. Strictly speaking, this definition differs from the definition first proposed by Minsky and Rosenblatt where the function spaces are of the form {0, ..., *n* − 1}^*k*^ → {0, 1}, which are binary classifiers.

**Definition 2**. A parallel machine Ψ : *X* → *Y* is a mathematical function of the form:


(2) $$\begin{array}{l} \text{Ψ}\left(\bar{x}\right)=\Omega \left(\varphi_{1}\left(\bar{x}\right),...,\varphi_{n}\left(\bar{x}\right)\right) \end{array}$$

where the functions of the finite set Φ = {φ_*i*_ : *X* → *Z*} are computed independently of each other and the function Ω : *Z*^*n*^ → *Y* is a function that combines all results with respect to some specification.

For practical implementations, it is necessary to mention that each function belonging to the set Φ will be a parametric function. That is, it will depend in at least one parameter per partial operator.

**Definition 3**. The perceptron is a learning structure capable of computing all linear functions in some given set Φ. The Rosenblatt perceptron is:


(3) $$\begin{array}{l} \text{Ψ}\left(\bar{x}\right)=\Omega \left(\varphi_{1}\left(\bar{x}\right),\varphi_{2}\left(\bar{x}\right),...\varphi_{n}\left(\bar{x}\right)\right)\\ \;=\overset{n}{\underset{i=1}{\sum }}\alpha_{i}\varphi_{i}\left(x_{1},...,x_{k}\right)>\theta \\ \;=\overset{n}{\underset{i=1}{\sum }}\alpha_{i}*x_{i}+b>\theta \end{array}$$

It is important to state that for the perceptron *n* = *k* + 1 where *k* is the arity of $\bar{x}$. In order to guarantee that each function $\varphi_{i}\left(\bar{x}\right)$ in the set Φ is linear, each φ_*i*_ defined can only depend on a particular coordinate from the vector $\bar{x}=\left(x_{1},...,x_{n}\right)$. The last term *b* which is defined by the partial operation $\varphi_{n}\left(\bar{x}\right)$ is a constant function called bias. The join function Ω is replaced by summing up all $\varphi_{n}\left(\bar{x}\right)$ and then applying an activation function which depends on the parameter θ. The threshold given by θ makes the perceptron behave as a binary classifier by outputting 1 if the sum is greater than θ, otherwise 0.

### 3.3. Signal Processing Background

The first functional implementations of the perceptron were mainly used to define filters as well as for equalization problems. Such are usually solved by signal processing methods (Wilson and Tufts, 1994; Kim and Adali, 2002). This was mainly due to its capabilities for automated learning and function approximation. Nonetheless, signal processing, creation of filters, and function approximation were always handled by Fourier analysis (Smith, 2010). Fourier analysis is a set of mathematical tools for signal processing; the most commonly used ones are Fourier series and Fourier transforms. The main idea of these tools is that periodic continuous functions can be decomposed into a (possibly infinite) sum of individual elements called sinusoids. Depending on the Fourier series definition (Smith, 2010), the sinusoids may either be real or complex functions. We will focus on the particular case where the function is defined as a sum of positive frequency complex sinusoids.

**Definition 4**. A complex sinusoid is a complex parametric function of the form:


(4) $$\begin{array}{l} s\left(x\right)=\alpha e^{i\omega x} \end{array}$$

where α ∈ ℂ defines the amplitude and phase of the signal^2^ and ω ∈ ℝ defines its frequency.

When the frequency of the complex sinusoid is positive, then it is called an analytic sinusoid. This is important for signal process analysis, as any real function can be encoded with only positive frequency sinusoids by simply generating a phase-quadrature component for each real sinusoidal by applying a Hilbert transform filter (Smith, 2010). This transformation allows us to reduce the amount of sinusoids required to define a real function by half, with the trade-off of using complex-valued functions. That is, amplitudes phases and frequencies will be defined by complex numbers.

**Figure 2:**
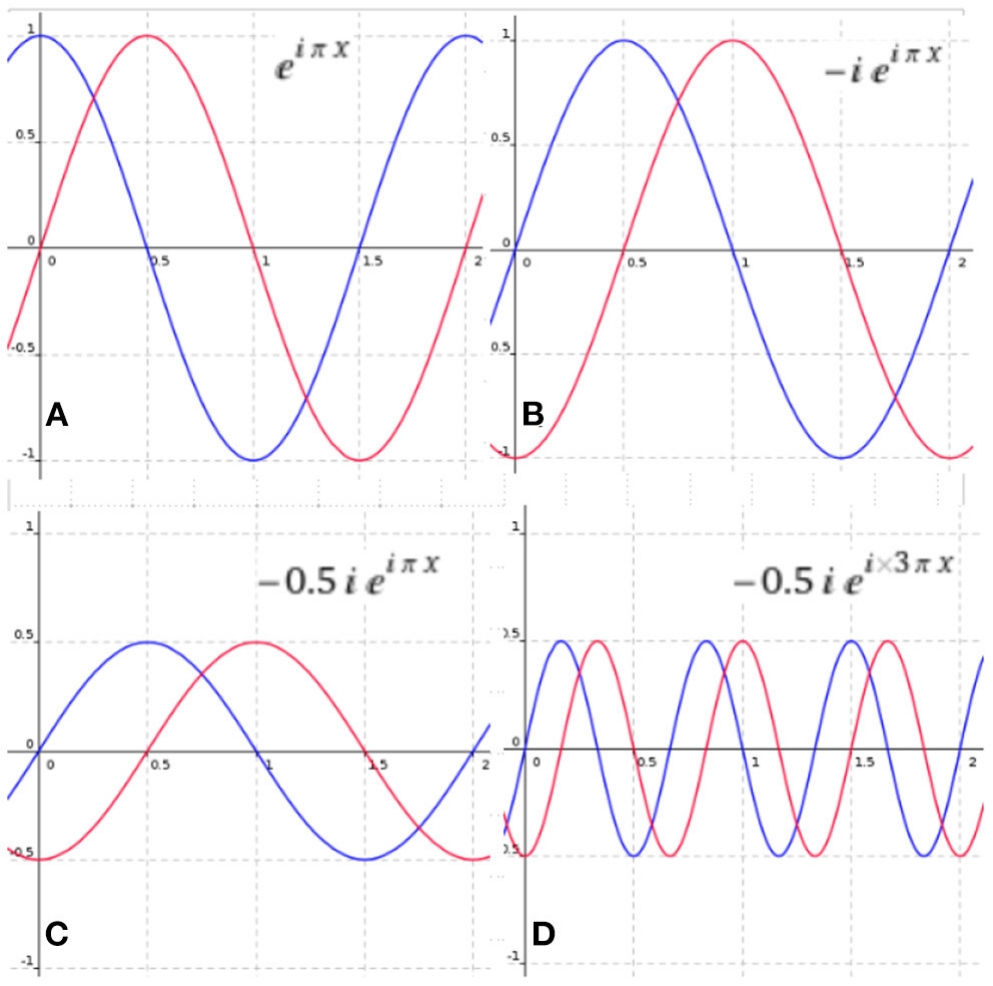
Modifying the parameters of an analytic sinusoid with amplitude 1 phase 0 and frequency π. The sinusoid in red represents the imaginary part of the complex sinusoid and the blue represents the real part. **(A)** The original signal, **(B)** the signal with a phase-shift, **(C)** the signal with a change of amplitude and phase-shift, and **(D)** a signal with a change of amplitude phase and frequency.

Figure 2 illustrates how an analytic sinusoid can be transformed by changing the complex parameter α, which will modify the amplitude and phase of the signal. It is important to notice that when α ∈ ℝ the only phase shift allowed is half the period. This can be achieved with negative real numbers. Thus, in signal processing there are only positive amplitudes since a negative amplitude will yield a phase shift of half the period of the signal.

Stating that, under the assumption that infinite sinusoids with different frequencies are provided, we can learn or define any analytic signal by finding the correct parameters for each sinusoid.

**Definition 5**. A *k*-dimensional analytic signal is a function that maps ℂ^*k*^ ⇒ ℂ. Any *k*-dimensional analytic signal can be expressed as an infinite sum of *k*-dimensional complex sinusoids:


(5) $$\begin{array}{l} s\left(\bar{x}\right)=\underset{\bar{\omega }\in {\mathbb{R}}^{k}}{\sum }\alpha_{\omega }e^{i\left(\bar{\omega }\cdot \bar{x}\right)} \end{array}$$

where $\bar{x}\in {\mathbb{C}}^{k}$ and α_ω_ ∈ ℂ.

Since Definition 5 assumes an infinite amount of sinusoids, the number of parameters α will also be infinite. The next section presents a new function learning method that overcomes this issue. Under the assumption of Section 3.1 that the function space is finite, our method can have a mathematical structure that adds a finite sum of signals to represent any function from a given finite function space.

## 4. Signal Perceptron

In this section, we introduce a new type of mechanism for learning functions that combines the structure of “parallel machines” with the type of functions used in signal processing into a unified method that we call **signal perceptron**. This section will discuss the original implementation and two variations of such parallel machines. We provide the theoretical definition, implementation, and analysis of these parallel machines. The analysis will be used to prove the expressiveness of our new methods against the perceptron (Rosenblatt, 1958) and the MLP defined in Sections 3.2 and 4.4, respectively. We will prove that signal perceptrons are not only able to learn complete Boolean functional spaces, but also that their space and computational complexity is smaller than the MLP, making them a more memory and computational efficient learning method.

### 4.1. Signal Perceptron Definitions

As discussed in Section 3.1 we are interested in implementations of parallel machines capable of learning any function from any function space. Since increasing the number of values in the domain/co-domain renders an exponential increase on the function space size, proofs and analyses of this section will be constrained to Boolean function spaces for the sake of exposition. We continue by defining the general form of a signal perceptron, where each partial operator is a fixed frequency analytic sinusoid. Each sinusoid depends on a parameter α ∈ ℂ, which defines the amplitude and phase of the sinusoid.

To give the definition of the signal perceptron we'll give an ordering to the set ***m***^***k***^ = {(*x*^0^, ..., *x*^*k*−1^) | *x*^*j*^ ∈ *m*} in the following way: for each *j* ∈ {0, ..., *m*^*k*^ − 1} we define $\bar{\omega_{j}}=\left(\omega_{j}^{0},...,\omega_{j}^{k-1}\right)$, where $\omega_{j}^{0}...\omega_{j}^{k-1}$ is the representation of *j* in base *m* of length *k* (in the Boolean case this means that this representation uses *k* bits).

**Definition 6**. A ***m***^***k***^ signal perceptron (SP) is a parametric function of the form,


(6) $$\begin{array}{l} s\left(\bar{x}\right)=\overset{m^{k}-1}{\underset{j=0}{\sum }}\alpha_{j}e^{\frac{i\pi }{m-1}\left(\bar{\omega_{j}}\cdot \bar{x}\right)} \end{array}$$

where *m, k* ∈ ℕ, $\bar{\omega_{j}}\in m^{k}$, α_*j*_ ∈ ℂ and $\bar{\omega_{j}}\cdot \bar{x}$ is the dot product.

**Figure 3:**
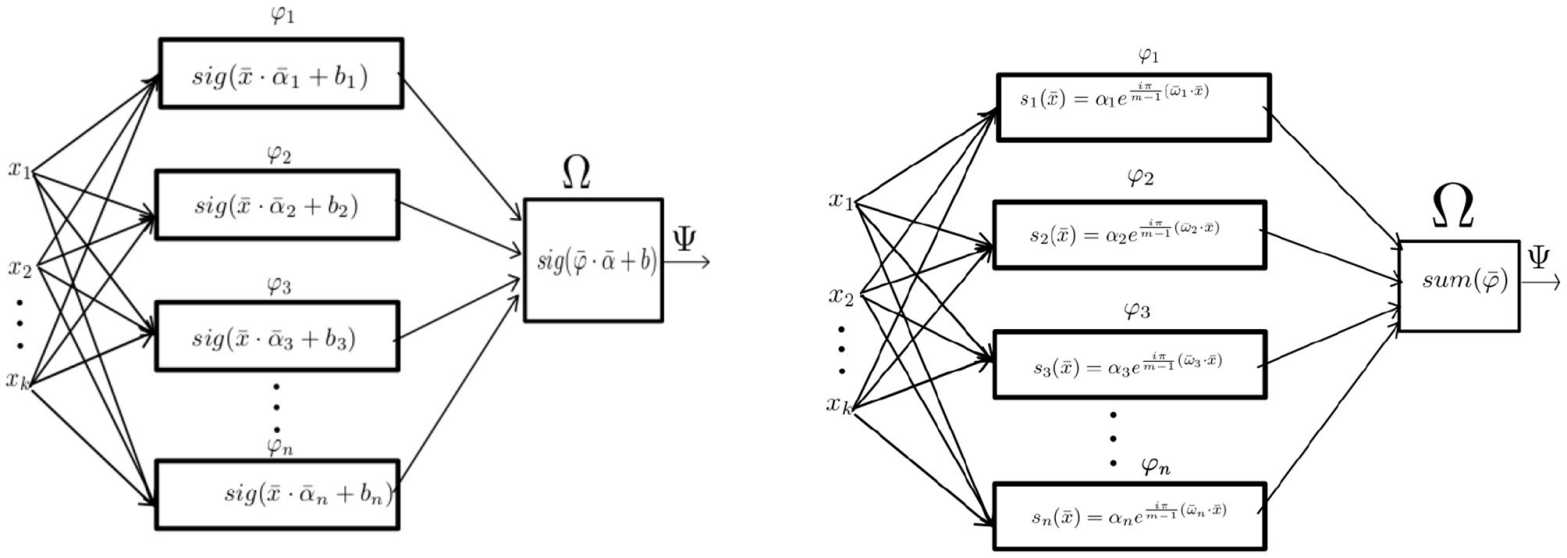
From left to right: The Implementation of the MLP with one hidden layer as a parallel machine and the implementation of the signal perceptron.

From Definition 6, we can deduce that the signal perceptron is a parametric parallel machine where each φ ∈ Φ is a *k*-dimentional analytic sinusoid and the function Ω is the sum operator. As shown in Figure 3, the amount of partial operators φ ∈ Φ for the signal perceptron is equal to the domain space size |***m***^***k***^| = *m*^*k*^. This number is the upper-bound of the amount of sinusoids required to generate any function of the function space Δ. That is, most of the functions from Δ will require less sinusoids to be defined. In other words, when obtaining the values of each α it is possible that some will be 0 making some partial operators unnecessary. Also, since the number of parameters coincides with the number of possible realizations of the domain size *m*^*k*^, the learning process of the α's can be obtained by treating each possible configuration of the inputs as a system of linear equations.

To better demonstrate such properties, Equation 6 can be reduced for the particular case where the functional space is the Boolean space, yielding the following:


(7) $$\begin{array}{l} s\left(\bar{x}\right)=\overset{2^{k}-1}{\underset{j=0}{\sum }}\alpha_{j}e^{i\pi \left(\bar{\omega_{j}}\cdot \bar{x}\right)} \end{array}$$

It's worth noticing that Equation 7 is the general definition for any *k*-ary Boolean function space. We can further expand this formula for the particular case of the binary Boolean function space, as shown in Example 1.

**Example 1.** By expanding Equation 7 for binary Boolean functions we obtain:


(8) $$\begin{array}{l} s\left(\bar{x}\right)=\alpha_{0,0}e^{i\pi \left(0*x_{2}+0*x_{1}\right)}+\alpha_{1,0}e^{i\pi \left(1*x_{2}+0*x_{1}\right)}\\ \;+\alpha_{0,1}e^{i\pi \left(0*x_{2}+1*x_{1}\right)}+\alpha_{1,1}e^{i\pi \left(1*x_{2}+1*x_{1}\right)} \end{array}$$

This gives us:


(9) $$\begin{array}{l} s\left(\bar{x}\right)=\alpha_{0,0}+\alpha_{1,0}e^{i\pi \left(x_{1}\right)}+\alpha_{0,1}e^{i\pi \left(x_{2}\right)}+\alpha_{1,1}e^{i\pi \left(x_{1}+x_{2}\right)} \end{array}$$

As shown in Algorithm 1 we can obtain the set of parameters α for a particular function, by defining a system of linear equations where each equation is a particular realization of the input variables. The process for obtaining the α's of a particular function is done by generating the signal perceptron's equation matrix as shown in Definition 7.

**Algorithm 1:** Learning algorithm for the signal perceptron based on a system of linear equations.

1: **Input:** batch of all $\bar{x}\in m^{k}$, vector equation *Y* that is the labels for each $\bar{x}\in m^{k}$. (i.e., objective function) Calculate the equation matrix $A_{m^{k}\times m^{k}}$ where for each element of the matrix *a*_*ij*_,$\bar{\omega },\bar{x}$ are the representation of *i, j* in base *m* of length *k*, respectively. *a*_*ij*_ is defined by replacing each $\bar{x}\in m^{k}$ into the signal perceptron equation: $a_{ij}=e^{\frac{i\pi }{m-1}\left(\bar{\omega }\cdot \bar{x}\right)}.$ Calculate inverse of matrix $A_{m^{k}\times m^{k}}$: ${A_{m^{k}\times m^{k}}}^{-1}$ Calculate vector of $\hat{\alpha }$: ${A_{m^{k}\times m^{k}}}^{-1}\cdot Y$

**Definition 7**. The signal perceptron learning method can be defined as a system of linear equations of the form: $\begin{array}{l} \left[\begin{array}{ccc} e^{\left(\frac{i\pi }{\left(m-1\right)}\left({\bar{\omega }}_{0}\cdot {\bar{x}}_{0}\right)\right)} & ... & e^{\left(\frac{i\pi }{\left(m-1\right)}\left({\bar{\omega }}_{m^{k}-1}\cdot {\bar{x}}_{0}\right)\right)}\\ . & \; & .\\ . & \; & .\\ . & \; & .\\ e^{\left(\frac{i\pi }{\left(m-1\right)}\left({\bar{\omega }}_{0}\cdot {\bar{x}}_{m^{k}-1}\right)\right)} & ... & e^{\left(\frac{i\pi }{\left(m-1\right)}\left({\bar{\omega }}_{m^{k}-1}\cdot {\bar{x}}_{m^{k}-1}\right)\right)} \end{array}\right]\;[\begin{array}{c} \alpha_{0}\\ .\\ .\\ .\\ \alpha_{m^{k}-1} \end{array}]=\left[\begin{array}{c} y_{0}\\ .\\ .\\ .\\ y_{m^{k}-1} \end{array}\right] \end{array}$

Each row of the matrix is defined by some vector ${\bar{x}}_{j}$ which is one possible realization of the domain. The vector $\bar{\alpha }$ represents the parameters to be learned and, the vector ȳ is the expected output of each possible realization of ${\bar{x}}_{j}$.

Then such matrix is multiplied by the vector of weights that defines the α's, which is equal to the vector ȳ which defines the function. The learning process amounts to calculate the inverse of the matrix and multiply it by the objective function ȳ to find the values of the α's that define such function.

An example of this implementation for the binary Boolean function space is illustrated in Table 2. We defined a signal perceptron as shown in Equation 7 and obtained the parameters of all binary Boolean functions which are depicted in Table 3.

**Table 3:** Table of all parameters required by the signal perceptron to define every binary Boolean function (as defined in Table 2).

**Function**	**α_1_, α_2_, α_3_, α_4_**	**Function**	**α_1_, α_2_, α_3_, α_4_**
0 0 0 0	0, 0, 0, 0	1 0 0 0	0.25, 0.25, 0.25, 0.25
0 0 0 1	0.25, –0.25, –0.25, 0.25	1 0 0 1	0.5, 0, 0, 0.5
0 0 1 0	0.25, 0.25, –0.25, –0.25	1 0 1 0	0.5, 0.5, 0, 0
0 0 1 1	0.5, 0, –0.5, 0	1 0 1 1	0.75 0.25, –0.25, 0.25
0 1 0 0	0.25, –0.25, 0.25, –0.25	1 1 0 0	0.5, 0, 0.5, 0
0 1 0 1	0.5, –0.5, 0, 0	1 1 0 1	0.75, –0.25, 0.25, 0.25
0 1 1 0	0.5, 0, 0, –0.5	1 1 1 0	0.75, 0.25, 0.25, –0.25
0 1 1 1	0.75, –0.25, –0.25, –0.25	1 1 1 1	1, 0, 0, 0

*These were obtained by running an implementation of Algorithms 1 and 2*.

It is important to note that in practice, it will be virtually impossible to define the signal perceptron matrices for bigger arities, as we will usually not have the output value for every possible domain realization. Nonetheless, the implementation depicted in Algorithm 1, is necessary in order to prove that the signal perceptron can learn any function for any *k*-ary Boolean function space as we will show in Section 4.2.

Before moving to such proof, we want to mitigate practical concerns regarding the learning of functions, using limited samples of the domain. That is, given a limited dataset we want to define an alternative learning algorithm that will allow the signal perceptron to approximate some function. For this reason, we define Algorithm 2, which allows us to overcome the need of providing the whole domain space by using gradient descent (Bottou et al., 2018).

**Algorithm 2:** Gradient descent based learning algorithm for the signal perceptron.

**Input:** *S* ⊆ *X* of ***m***^***k***^ domain and labels *Y*, vector of weights $\hat{\alpha }$, learning rate γ, number of iterations *iter* Generate signal perceptron for *m*^*k*^space:$s_{m^{k}}\left(\bar{x}\right)$
**For** *i* **in** *iter*: Calculate the gradient of loss $\nabla f\left({\hat{\alpha }}_{old}\right)$ Assuming loss is MSE: $\nabla f\left({\hat{\alpha }}_{old}\right)=\left[\begin{array}{c} \frac{df}{d\alpha_{1}}\\ .\\ .\\ .\\ \frac{df}{d\alpha_{n}} \end{array}\right]\left[\begin{array}{c} \frac{-2}{N}\sum_{\bar{x}\in S}e^{i\pi \bar{\omega_{1}}\cdot \bar{x}}\left(y-s_{m^{k}}\left(\bar{x}\right)\right)\\ .\\ .\\ .\\ \frac{-2}{N}\sum_{\bar{x}\in S}e^{i\pi \bar{\omega_{n}}\cdot \bar{x}}\left(y-s_{m^{k}}\left(\bar{x}\right)\right) \end{array}\right]$ Update the weights: ${\hat{\alpha }}_{new}={\hat{\alpha }}_{old}-\gamma \nabla loss\left({\hat{\alpha }}_{old}\right)$
2: **Output:** $\hat{\alpha }$

Interestingly, just as Algorithm 1, the implementation of Algorithm 2 allowed us to learn the same parameters depicted in Figure 3 when learning all functions from the binary Boolean function space.^3^ An in-depth analysis of the hyperparameters used to solve this task is discussed in Section 5.

### 4.2. Signal Perceptron: A Universal Boolean Function Learner

Following the definition of Algorithm 1, we will now proceed with the proof that the perceptron is a universal Boolean learning machine. This is based on the theorem for the general signal perceptron formula of Equation 6:

**Proposition 1**. *For all functions of the form f* : *m*^*k*^ → *m*^1^, *there exists a set of* $\alpha_{\omega }\in C^{m^{k}}$ *such that*


$$f\left(\bar{x}\right)=\underset{\bar{\omega }\in m^{k}}{\sum }\alpha_{\omega }e^{\frac{i\pi \left(\bar{\omega }\cdot \bar{x}\right)}{m-1}}$$

In other words, Proposition 1 states that any function from any functional space ***m***^***k***^ can be expressed using the signal perceptron parametric function. As stated before, we are interested in the Boolean space where logic connectives reside. Due to this, we will prove Proposition 1 only for the Boolean case, that is for all *k*-ary Boolean functions, which will be defined as the Theorem:

**Theorem 1**. *For all k-ary Boolean functions, f* : 2^*k*^ → 2, *there exists a set of complex coefficients* ${\left\{\alpha_{{\bar{\omega }}_{j}}\right\}}_{{\bar{\omega }}_{j}\in 2^{k}}$ *such that:*


(10) $$\begin{array}{l} f\left(\bar{x}\right)=\overset{2^{k}-1}{\underset{j=0}{\sum }}\alpha_{\bar{\omega_{j}}}e^{i\pi \left(\bar{\omega_{j}}\cdot \bar{x}\right)} \end{array}$$

*Proof*. Let *f* : 2^*k*^ → 2 be any *k*-ary Boolean function. In order to find the coefficients $\left\{\alpha_{\bar{\omega_{j}}}\right\}$ from Equation 10, we have to solve the system of equations


$${\left\{s\left(\bar{\omega_{j}}\right)=f\left(\bar{\omega_{j}}\right)\right\}}_{j=0}^{2^{k}-1}$$

This system has a unique solution if and only if the coefficient matrix


(11) $$\begin{array}{l} A_{k}={\left(e^{i\pi \bar{\omega_{j}}\cdot {\bar{\omega }}_{l}}\right)}_{j,l}={\left({\left(-1\right)}^{\bar{\omega_{j}}\cdot {\bar{\omega }}_{l}}\right)}_{j,l}\text{ where }j,l\in \left\{0,...,2^{k}-1\right\} \end{array}$$

is invertible. We will prove this by showing that the matrices are of the form


$$A_{k+1}=\left(\begin{array}{cc} A_{k} & A_{k}\\ A_{k} & -A_{k} \end{array}\right)$$

which means that they are precisely the Walsh matrices. This family of matrices are also Hadamard matrices Kanjilal (1995), which satisfy that $A_{k}A_{k}^{t}=A_{k}^{t}A_{k}=2^{k}I$, i.e., they are orthogonal and in particular invertible. We will proceed to prove this by diving the matrix in 4 blocks


$$A_{k+1}=\left(\begin{array}{cc} A & B\\ C & D \end{array}\right)$$

where each block is a 2^*k*^ × 2^*k*^ matrix and prove that *A*_*k*_ = *A* = *B* = *C* = −*D*. Let's denote *A*_*k*_ = (α_*jl*_) and define the matrices in the following way. For all 0 ≤ *j, l* ≤ 2^*k*^ − 1:


$$\begin{array}{l} A=\left(a_{jl}\right):=\left(\alpha_{jl}\right)=\left({\left(-1\right)}^{{\bar{\omega }}_{j}\cdot {\bar{\omega }}_{l}}\right)\\ B=\left(b_{jl}\right):=\left(\alpha_{j\left(l+2^{k}\right)}\right)=\left({\left(-1\right)}^{{\bar{\omega }}_{j}\cdot {\bar{\omega }}_{\left(l+2^{k}\right)}}\right)\\ C=\left(c_{jl}\right):=\left(\alpha_{\left(j+2^{k}\right)l}\right)=\left({\left(-1\right)}^{{\bar{\omega }}_{\left(j+2^{k}\right)}\cdot {\bar{\omega }}_{l}}\right)\\ D=\left(d_{jl}\right):=\left(\alpha_{\left(j+2^{k}\right)\left(l+2^{k}\right)}\right)=\left({\left(-1\right)}^{{\bar{\omega }}_{\left(j+2^{k}\right)}\cdot {\bar{\omega }}_{\left(l+2^{k}\right)}}\right) \end{array}$$

The order of the matrix is given by expressing the numbers in base 2. Due to this, for each ${\bar{\omega }}_{j}$, if *j* is greater or equal than 2^*k*^ then the last coordinate of ${\bar{\omega }}_{j}$ will be 1. Otherwise it will be 0 i.e.,


(12) $$\begin{array}{r} 0\leq j\leq 2^{k}-1\;\Leftrightarrow \;\omega_{j}^{k+1}=0\\ 2^{k}\leq j\leq 2^{k+1}-1\;\Leftrightarrow \;\omega_{j}^{k+1}=1 \end{array}$$

These equivalences tell us that when *j* ≤ 2^*k*^ − 1 or *l* ≤ 2^*k*^ − 1 the last coordinate of ${\bar{\omega }}_{j}$ will be 0. For this reason, the inner product $\left(\omega_{j}^{1},...,\omega_{j}^{k}\right)\cdot \left(\omega_{l}^{1},...,\omega_{l}^{k}\right)$ of a representation of arity *k* will be the same as that of a representation of arity *k* − 1 ($\left(\omega_{j}^{1},...,\omega_{j}^{k-1}\right)\cdot \left(\omega_{l}^{1},...,\omega_{l}^{k-1}\right)$). If 2^*k*^ ≤ *j* and 2^*k*^ ≤ *l* then $\omega_{j}^{k+1}\omega_{l}^{k+1}=1$. Consequently, the inner product ${\bar{\omega }}_{j}\cdot {\bar{\omega }}_{l}$ will be $\left(\omega_{j}^{1},...,\omega_{j}^{k-1}\right)\cdot \left(\omega_{l}^{1},...,\omega_{l}^{k-1}\right)+1$ i.e.,


(13) $$\begin{array}{l} {\bar{\omega }}_{j}\cdot {\bar{\omega }}_{l}=\{\begin{array}{ll} \overset{k}{\underset{n=1}{\sum }}\omega_{j}^{n}\omega_{l}^{n} & \text{if }0\leq j\leq 2^{k}-1\\ \; & \text{or }0\leq l\leq 2^{k}-1\\ \overset{k}{\underset{n=1}{\sum }}\omega_{j}^{n}\omega_{l}^{n}+1 & \text{if }2^{k}\leq j,l\leq 2^{k+1}-1 \end{array} \end{array}$$

Since for all 0 ≤ *i* ≤ 2^*k*^ − 1 we have


$${\bar{\omega }}_{i+2^{k}}=\left(\omega_{i}^{1},...,\omega_{i}^{k},1\right)$$

using 12 and 13 we obtain the following equations for all 0 ≤ *j, l* ≤ 2^*k*^ − 1,


$$\overset{k}{\underset{n=1}{\sum }}\omega_{j}^{n}\omega_{l}^{n}=\overset{k+1}{\underset{n=1}{\sum }}\omega_{j}^{n}\omega_{l}^{n}={\bar{\omega }}_{j}\cdot {\bar{\omega }}_{l}={\bar{\omega }}_{j}\cdot {\bar{\omega }}_{\left(l+2^{k}\right)}={\bar{\omega }}_{j+2^{k}}\cdot {\bar{\omega }}_{l}$$

by definition of *A, B, C* this implies that


$$\alpha_{jl}=a_{jl}=b_{jl}=c_{jl}.$$

So *A*_*k*_ = *A* = *B* = *C*

Using Equation 13 for all 0 ≤ *j, k* ≤ 2^*k*^ − 1


$${\bar{\omega }}_{\left(j+2^{k}\right)}\cdot {\bar{\omega }}_{\left(l+2^{k}\right)}={\bar{\omega }}_{j}\cdot {\bar{\omega }}_{l}+1$$

we conclude that


$$\begin{array}{c} d_{jl}=\left({\left(-1\right)}^{{\bar{\omega }}_{\left(j+2^{k}\right)}\cdot {\bar{\omega }}_{\left(l+2^{k}\right)}}\right)=\left({\left(-1\right)}^{{\bar{\omega }}_{j}\cdot {\bar{\omega }}_{l}+1}\right)\\ =-\left({\left(-1\right)}^{{\bar{\omega }}_{j}\cdot {\bar{\omega }}_{l}}\right)=-\alpha_{jl} \end{array}$$

so *D* = −*A*_*k*_

this proves that


$$A_{k+1}=\left(\begin{array}{cc} A_{k} & A_{k}\\ A_{k} & -A_{k} \end{array}\right)$$

Which means that the matrices *A*_*k*_ are precisely Walsh matrices of dimension 2^*k*^. This completes the proof that the matrices are indeed invertible for all *k*.

As shown by the proof of Theorem 1, the type of matrices that the *k*-ary Boolean signal perceptron generates are Walsh matrices. Example 2 provides the Walsh matrices used to calculate the parameters of any unary and binary Boolean functions.

**Example 2**. Equation matrices *A*_2**x**2_, *A*_4**x**4_ generated by the signal perceptron for solving any unary and binary Boolean function, respectively. The real part of such complex matrices are also called Walsh matrices which are a particular case of the family of matrices called Hadamard matrices. The complex matrices can also be defined with two real valued matrices, the first matrix representing the real part and the other imaginary part as showed for *A*_4**x**4_.


$$\begin{array}{c} A_{2\text{x}2}=\left[\begin{array}{cc} 1+0i & \;1+0i\\ 1+0i & -1+0i \end{array}\right]\\ A_{4\text{x}4}=\left[\begin{array}{cccc} 1+0i & \;1+0i & \;1+0i & \;1+0i\\ 1+0i & -1+0i & \;1+0i & -1+0i\\ 1+0i & \;1+0i & -1+0i & -1+0i\\ 1+0i & -1+0i & -1+0i & \;1+0i \end{array}\right]\\ A_{4\text{x}4}=A+iB=\left[\begin{array}{cccc} 1 & \;1 & \;1 & \;1\\ 1 & -1 & \;1 & -1\\ 1 & \;1 & -1 & -1\\ 1 & -1 & -1 & \;1 \end{array}\right]+i\;[\begin{array}{cccc} 0 & \;0 & \;0 & \;0\\ 0 & \;0 & \;0 & \;0\\ 0 & \;0 & \;0 & \;0\\ 0 & \;0 & \;0 & \;0 \end{array}] \end{array}$$

In such example,it is shown that we can transform the complex matrix into two real valued matrices, one representing the real part while the other the imaginary part. This interpretation is important because analytic signals can be also defined in terms of two real functions, one representing the real part and the second the imaginary part of the complex function using the identity:


(14) $$\begin{array}{l} s\left(x\right)=\alpha e^{i\omega x}=\alpha cos\left(\omega x\right)+i\alpha sin\left(\omega x\right) \end{array}$$

The identity in Equation 14 is only possible when the input *x* is a real number. Since for Boolean function spaces that assumption is always true, we can observe that the real part of the matrix generated by the signal perceptron is defined only by the function α*cos*(ω*x*). Because of this, we can define a variation of the signal perceptron which is defined only by the real part which will be discussed in the next section.

### 4.3. Signal Perceptron Variations

As explained above, the Walsh matrix in the complex domain is the matrix for the real part which contains all the information to define Boolean function spaces. For this reason, we will proceed to define a signal perceptron that works only within the real domain.

**Definition 8**. The Real Signal Perceptron (RSP) is a parallel machine of the form:


(15) $$\begin{array}{l} s\left(\bar{x}\right)=\overset{m^{k}-1}{\underset{j=0}{\sum }}\alpha_{j}cos\left(\frac{\pi }{m-1}\bar{\omega_{j}}\cdot \bar{x}\right) \end{array}$$

where *m, k* ∈ ℕ, $\bar{\omega_{j}}\in m^{k}$, α_*j*_ ∈ ℝ and $\bar{\omega_{j}}\cdot \bar{x}$ is the dot product.

From Definition 8, RSP can be understood as a less general variation of the signal perceptron. Interestingly, this definition is sufficient to guaranty that we can learn any function of the Boolean space as explained at the end of the previous section. While at first glance this new signal perceptron may seem a limited and less expressive implementation, such variation has some practical advantages over the original signal perceptron. Some of the current deep learning libraries (e.g., PyTorch and TensorFlow), lack the capabilities of dealing with learnable parameters and functions defined in the complex domain. Thus, by limiting our signal perceptron to the real domain, we can directly integrate it in most deep learning libraries, which would allow us to take advantage of all preexisting gradient descent optimizers, loss functions and other deep learning functions as we will show in Section 5.

In practice, such libraries are used to learn a particular function from a given function space. As mentioned previously, both the definitions of the signal perceptron and the RSP, provide an upper bound to the amount of signals necessary to represent the entirety of the functional space. Consequently, in order to define a particular function, the amount of signals will be less or equal than the upper bound.

We will next introduce a variation of the RSP that can work with a variable number of signals. We call this variation the Fourier signal perceptron and it is defined as:

**Definition 9**. The Fourier Signal Perceptron (FSP) is a parallel machine where the amplitudes and frequencies of the signals are learnable parameters.


(16) $$\begin{array}{l} s\left(\bar{x}\right)=\overset{n-1}{\underset{j=0}{\sum }}\alpha_{j}cos\left(\bar{\omega_{j}}\cdot \bar{x}\right) \end{array}$$

where $\bar{\omega_{j}}$, α_*j*_ ∈ ℝ and $\bar{\omega_{j}}\cdot \bar{x}$ is the dot product.

As shown in Definition 9, the important difference between the FSP and the RSP is the amount of signals. While the RSP requires *m*^*k*^ signals, the FSP can be defined using any amount. This is possible since the frequencies are now learnable parameters. For the particular case when the amount of signals of the FSP equals to *m*^*k*^, Definition 9 becomes the same as the RSP one, assuming that the same frequencies are used. With this simple change, we can create a powerful function approximator that can be used for continuous domains. As we will show in Section 5, the FSP can be used straightforwardly to learn image classification tasks without having the issue of an exponential blowup in the amount of parameters required to learn all functions.

### 4.4. The Multilayer Perceptron vs. the Signal Perceptron

Before we continue with the experiments of Section 5, we will briefly analyze the differences between the single hidden layer MLP and the signal perceptron (SP). This is important as it will provide clarity to better understand why the SP outperforms the MLP in all experiments conducted. A similar analysis is conducted in Section 6.2 for other literature neurons that claim to solve the non-linear separability problem.

As shown in Figure 3, the single hidden layer MLP can be defined in terms of a parallel machine as:

**Definition 10**. A MLP with one hidden layer is a parallel machine, where each partial operator $\varphi_{1}\left(\bar{x}\right),..,\varphi_{n}\left(\bar{x}\right)$ is a perceptron and the join function Ω is also a perceptron.

That is:


(17) $$\begin{array}{l} \text{Ψ}\left(\bar{x}\right)=\Omega \left(\varphi_{1}\left(\bar{x}\right),\varphi_{2}\left(\bar{x}\right),...\varphi_{n}\left(\bar{x}\right)\right)\\ \;=\Omega \left(\overset{k}{\underset{i=1}{\sum }}\alpha_{1,i}*x_{i}+b_{1}>\theta_{1},...,\overset{k}{\underset{i=1}{\sum }}\alpha_{n,i}*x_{i}+b_{n}>\theta \right)\\ \;=\overset{n}{\underset{j=1}{\sum }}\left(\overset{k}{\underset{i=1}{\sum }}\alpha_{j,i}*x_{j}+b_{j}>\theta_{j}\right)>\theta \end{array}$$

From Definition 10, the amount of perceptrons in the 'hidden layer' to learn any function, is equivalent to the training samples which is *n* = *m*^*k*^ as explained by Huang and Babri (1997) and Baum (1988). Which means that the total amount of perceptrons for the whole structure is equal to *m*^*k*^ + 1. Where *m*^*k*^ perceptrons are used to represent the partial operators and the last perceptron represents the Ω function. It is worth noticing, that for the SP and RSP (as shown in Definitions 6,8), the amount of partial operators required remains the same as the 'hidden layer' of the MLP, which is *m*^*k*^.

The main difference will reside in the amount of learnable parameters, which for the case of the MLP, the amount of parameters required to learn any function is (*k* + 1)*n* + *n* + 1 = (*k* + 2)*n* + 1, where *n* = *m*^*k*^.

On the other hand, as depicted in Figure 3, the only learnable parameters are the amplitudes of each signal. This makes the spatial complexity *n* if the complex numbers are counted as one parameter, or 2*n* if the complex number is thought as two separated real numbers representing the real and imaginary part, respectively.

For the case of the RSP the amount of parameters is *n* and for the FSP is *kn* which are always smaller than the MLP.

Regarding the structural analysis, the amount of linear and non-linear operations is almost the same for both MLP and SP variations. As shown in Definitions (6,8,9), the operations for the three SP variations remains the same. First a dot product between the frequencies and input vectors, then applying the non-linear operation, which is a real or complex sinusoid. The final operation is the dot product of the vector of signals by the vector of α's. This amounts to a total of *n* + 1 linear operations and *n* non-linear operations, where *n* is the amount of partial operators.

For the case of the MLP, based on Definition 10, we will have the following hidden layer operations. A dot product between the weights and input vectors, following addition of the vector of bias then applying the non-linear operation, which could contain extra linear operations, depending on the activation function. Then for the second layer, the operations are: a dot product between the output of the hidden layer and the weights of the output layer, an addition of the bias and finally applying the non-linear operation, which could contain extra linear operations depending on the activation function. This give us a total of *n* + 1 linear operations and n non-linear operations for the first layer, and 2 linear operations plus 1 non linear operation in the second layer. This results into a total of *n* + 2 linear and *n* + 2 non-linear operations, assuming that the activation function contains only one non-linear operation. Such assumption is usually broken, since activation functions tend to have multiple linear and non-linear operations. A common example is the sigmoid function, which contains one linear operation (division) and one non-linear operation (exponentiation). This will increase the total number of operations to 2*n* + 2 linear and *n* + 2 nonlinear.

Finally, signal perceptrons are mechanisms that are able to learn all functions exactly, given the whole domain as training samples. At least, for the *k*-ary Boolean function space this was proven analytically. In contrast, the MLP was only proven analytically (Baum, 1988) to be a function approximator, rather than an exact function learner. Because of this, such mechanism require lots fine tuning of hyperparameters, specific choice of activation functions and restructuring its topology to achieve the same goal. That is not the case for the signal perceptron which we showed it doesn't require fine tuning and solve the tasks without the need of optimizers. Even for the case that the training samples are limited, signal perceptrons can be used as function approximators, which outperformed the generalization capabilities of MLP. This is shown in the experimental evaluation in Section 5.

## 5. Experiments

Now we proceed to demonstrate experimentally the signal perceptron's potential advantages over the one hidden layer MLP. As stated previously, we are interested in mechanisms able to learn all functions within a functional space with just one structure. For this reason, our first set of experiments will be focused on performing this task over the binary Boolean function space as shown in Table 2. We will evaluate implementations of the SP, RSP, FSP and the single hidden layer MLP, by measuring the amount of learnable parameters required for each structure to learn all functions from the functional space, the average time that each method requires for inference and training, and finally their learning capabilities. These are measured by the average loss at the end of the training.

For the second set of experiments we measured the learning capabilities of such structures when learning all functions simultaneously. The 16 functions of the binary Boolean function space can be learned simultaneously by defining a signal perceptron of 16 outputs. This is possible due to Definitions 6, 8, and 9, which show that all functions from the functional space are sharing the same frequencies. Since the amplitudes are the ones defining each function from the functional space, we can exploit this property by defining a signal perceptron of *n*-vectors of amplitudes. To compare learning performance against the single hidden layer MLP, we defined the MLP's last layer by using *n*-nodes rather than a single node^4^.

The third part of the experiments aims at highlighting the potential practical uses of the signal perceptron. We will focus our attention into learning two different image classification datasets MNIST and FashionMNIST (LeCun and Cortes, 2010; Xiao et al., 2017) using different FSP and MLP architectures. This is done in order to assess the performance of signal perceptrons in function approximation and generalization when trained with limited data.

### 5.1. Evaluation

For our first set of experiments, we defined an architecture that is only able to learn one function from the binary Boolean function space. Because of this, the training process for learning all functions was to use the same initial learnable parameters, every time the training algorithm starts over to learn a new function from the the binary Boolean function space. Finally we conducted the experiments a total of five times and averaged the results to in order to prevent single training bias.

In order to provide a fair comparison against the MLP with one hidden layer we will work with the following criteria:

- The MLP used by these experiments will be constructed as shown in Definition 10 and is based on the analytic proof given by Huang and Babri (1997). That is, our MLP will use sigmoid activation functions and the number of perceptrons used in the hidden layer will be equal to the number of training samples. For the binary Boolean function space that is: *m*^*k*^ = 2^2^ = 4 nodes.
- The FSP variation will use the same amount of signals as the SP and the RSP, which is equal to 4.

To evaluate spatial and sample complexity we measure the amount of learnable parameters, the average forward time for inference and average backward time during training, leading to the results illustrated in Table 4. The signal perceptron and the RSP implementations are the ones with the less amount of parameters as well as the ones taking less average inference and training runtime. This is mainly due to the fact that the frequencies of the signals are not learnable parameters. In contrast, the FSP and MLP take more runtime because of the extra set of parameters required to learn all the functions. Still, it is demonstrated that the parameters required for the FSP and runtimes are more optimal than the MLP ones. It is important to mention that we had to code our own NumPy implementation of the signal perceptron as the current version of PyTorch cannot operate properly with complex functions and parameters.

**Table 4:** Spatial complexity and learning times of different architectures.

**Country list**					
**Property**	**SP NumPy**	**RSP NumPy**	**RSP PyTorch**	**FSP PyTorch**	**MLP_***h*1**_ PyTorch**
Learnable parameters	4	4	4	12	17
Avg forward/inference time (ms)	0.0085	0.0065	0.0409	0.0270	0.0410
Avg backward/backprop time (ms)	0.0409	0.0336	0.1698	0.1899	0.2232

*Signal perceptron (SP) NumPy, real signal perceptron (RSP) NumPy, RSP PyTorch, Fourier signal perceptron (FSP) PyTorch, and single hidden layer MLP PyTorch*.

All the architectures were trained using batch gradient descent with no dropout regularization (Srivastava et al., 2014) and the mean squared error (MSE) loss function. The dataset used for training consisted of all possible realizations of the domain as depicted in Table 2. The training analysis was made by running multiple times the training algorithm with different numbers of epochs (100, 1,000, 10,000, 20,000) and learning rates (0.1, 0.01, 0.001), which yielded the results illustrated in Tables 5, 6.

**Table 5:** Number of learned functions per method when learning the sixteen functions of the binary Boolean function Space.

**Epochs**	**LR**	**SP NumPy**	**RSP NumPy**	**RSP PyTorch**	**FSP PyTorch**	**MLP_***h*1**_ PyTorch**
100	0.001	0	0	0	0	0
100	0.01	0	0	0	0	0
100	0.1	16	16	16	16	1
1,000	0.001	0	0	0	0	0
1,000	0.01	16	16	16	2	0
1,000	0.1	16	16	16	16	0
10,000	0.001	16	16	16	1	0
10,000	0.01	16	16	16	16	0
10,000	0.1	16	16	16	16	6
20,000	0.001	16	16	16	9	0
20,000	0.01	16	16	16	16	2
20,000	0.1	16	16	16	16	14

*The methods compared are different implementations of the signal perceptron and the MLP with one hidden layer*.

**Table 6:** Average final loss for different implementations of the signal perceptron and MLP with one hidden layer when learning the sixteen functions of the binary Boolean function space.

**Epochs**	**LR**	**SP NumPy**	**RSP NumPy**	**RSP PyTorch**	**FSP PyTorch**	**MLP_***h*1**_ PyTorch**
100	0.001	0.6233 − 1.8718*j* · 10^−17^	0.7238	0.4258	0.2629	0.2476
100	0.01	0.0718 + 2.3059*j* · 10^−17^	0.0189	0.0164	0.1776	0.2260
100	0.1	4.2005 · 10^−20^ + 1.7220*j* · 10^−34^	9.4023 · 10^−20^	5.7768 · 10^−15^	0.0566	0.1868
1,000	0.001	0.6233 − 1.8718*j* · 10^−17^	0.7238	0.4258	0.2629	0.2476
1,000	0.01	1.7142 · 10^−18^ + 4.8695*j* · 10^−33^	2.1990 · 10^−18^	1.4449 · 10^−12^	0.0656	0.1864
1,000	0.1	2.5458 · 10^−32^ + 2.4818*j* · 10^−48^	3.4570 · 10^−32^	7.2082 · 10^−15^	5.1609 · 10^−05^	0.1264
10,000	0.001	2.2651 · 10^−18^ + 1.2682*j* · 10^−32^	5.7428 · 10^−18^	1.4452 · 10^−10^	0.0535	0.1865
10,000	0.01	5.6787 · 10^−30^ + 8.6296*j* · 10^−46^	5.0416 · 10^−30^	1.2703 · 10^−12^	2.4053 · 10^−05^	0.1050
10,000	0.1	3.0393 · 10^−32^ + 6.0275*j* · 10^−48^	5.7537 · 10^−32^	6.9512 · 10^−15^	8.8542 · 10^−08^	0.0264
20,000	0.001	5.7354 · 10^−28^ + 3.5035*j* · 10^−44^	6.0837 · 10^−28^	1.6507 · 10^−10^	0.0040	0.1806
20,000	0.01	5.3189 · 10^−30^ + 1.4378*j* · 10^−46^	4.6655 · 10^−30^	1.6408 · 10^−12^	1.2077 · 10^−05^	0.0536
20,000	0.1	2.7013 · 10^−32^ + 5.8603*j* · 10^−48^	3.7074 · 10^−32^	6.5677 · 10^−15^	2.8152 · 10^−08^	0.0012

From Table 5, we can observe the amount of functions of the binary Boolean function space learned by each structure. We use a threshold of 0.001 to consider that a structure learned a function to a satisfying degree. This threshold was applied to the final loss at the end of training.

**Figure 4:**
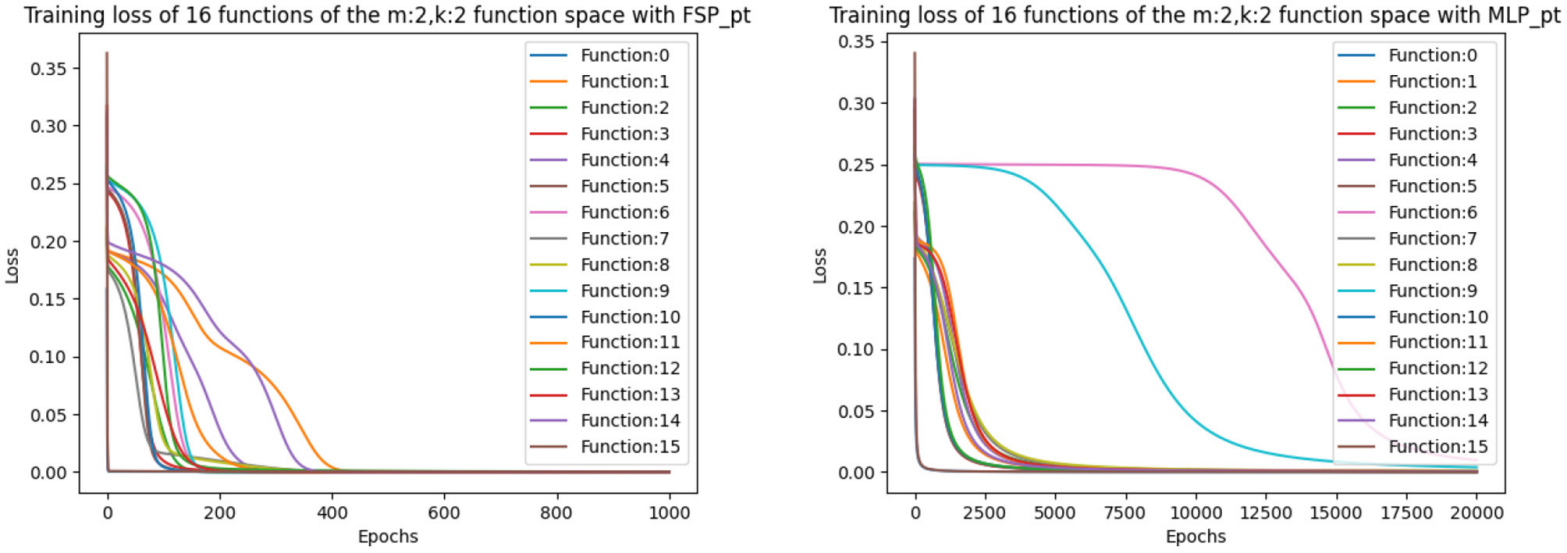
Figure that plots the loss function of all 16 binary Boolean functions using the FSP PyTorch and the MLP PyTorch implementation using learning rate of 0.1. The graph on the right shows that even after 20,000 epochs, the MLP cannot exactly learn some of the functions.

For a more in-depth analysis of the hyperparameters, we averaged over the final loss of the 16 possible functions. The results are displayed in Table 6. From Table 6, we can observe that the optimal learning rate for the SP and RSP is 0.1, since using smaller learning rates takes the models more epochs to converge. For the FSP, the optimal learning rate is also 0.1 but it requires more epochs to converge as it needs to learn the frequencies of the signals. Meanwhile, for the MLP there is no optimal learning rate, as it never achieves the loss threshold of less than 0.001. Not even when the amount of epochs is equal to 20,000. This happens because there are two functions from the Boolean function space that the MLP is never able to learn. These functions can be observed in Figure 4, being number 6 and 9, namely the *XOR* and not-*XOR*, respectively. By using the ADAM optimizer, the MLP can learn the functions in nearly 10000 epochs which are still more than using any signal perceptron with gradient descent^5^. While in the case of the signal perceptron variations, using the ADAM optimizer required less than 200 epochs to learn all functions.

The second set of experiments requires to learn all 16 functions that define the binary Boolean function space simultaneously. Just as in the first set of experiments, we evaluated the spatial complexity by measuring the amount of learnable parameters, then we also calculate the average forward and the average backward time, as shown in Table 7.

**Table 7:** Spatial complexity and learning times of different architectures for learning in parallel all 16 functions from the Binary Boolean Function Space for different implementations of the signal perceptron and MLP with one hidden layer.

**Property**	**SP NumPy**	**RSP NumPy**	**RSP PyTorch**	**FSP PyTorch**	**MLP_***h*1**_ PyTorch**
Learnable parameters	64	64	64	72	92
Avg forward/inference time (ms)	0.0089	0.0066	0.0412	0.0273	0.0349
Avg backward/backprop time (ms)	0.0420	0.0323	0.1808	0.2238	0.2360

In Table 7, we can observe an increment of parameters in the signal variations and MLP. This is due to the multiple vectors of α's and multiple nodes (for the MLP last layer), required to learn all functions at once. Nonetheless, this table follows the same pattern as Table 4 where all SP variations outperformed the MLP for every metric used.

**Figure 5:**
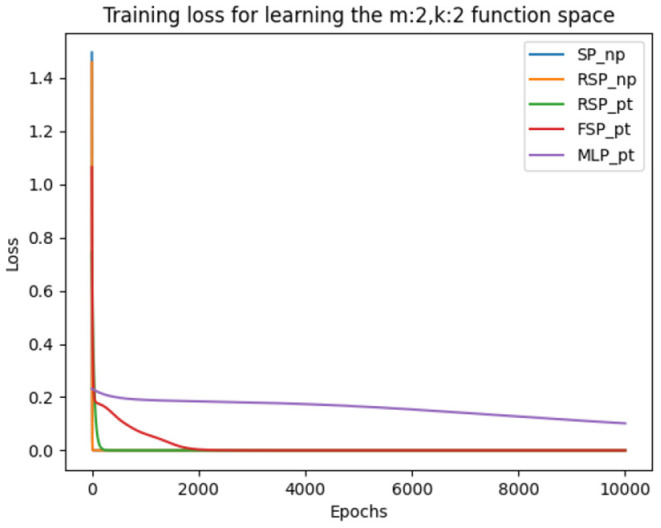
Figure that plots the loss function of different architectures when learning the whole binary Boolean space simultaneously with batch gradient descent for 10,000 episodes and learning rate of 0.1.

During training we used the same hyperparameters, loss function (MSE) and training method (batch gradient descent) as in the previous experiments. The results depicted in Table 8 are the average of the five runs conducted in order to ensure consistency and the precision of our measurements. We can observe a similar learning pattern as in the first set of experiments, where the optimal learning rate is also 0.1 for all variations of the signal perceptron. Indeed, the amount of epochs to achieve the loss error threshold of 0.001 increases, but this is because of learning multiple tasks in parallel. We discovered that the MLP encounters the same problem as in the previous experiment and is not able to learn some of the functions. This can be observed in Figure 5, where every signal perceptron variation achieves zero loss in less than 4000 epochs and the MLP is stuck at a loss of 0.2 even after 10,000 epochs.

**Table 8:** Measuring final loss when learning all 16 functions of the binary Boolean function Space, using different implementations of the signal perceptron and MLP with one hidden layer.

**Epochs**	**LR**	**SP NumPy**	**RSP NumPy**	**RSP PyTorch**	**FSP PyTorch**	**MLP PyTorch**
100	0.001	1.2122 − 3.9221*j* · 10^−17^	0.9694	0.7269	0.8520	0.2357
100	0.01	0.0262 + 1.0550*j* · 10^−17^	0.0320	0.5925	0.3650	0.2647
100	0.1	1.0533 · 10^−19^ + 4.8589*j* · 10^−34^	8.5732 · 10^−20^	0.0828	0.1835	0.2254
1,000	0.001	1.2122 − 3.9221*j* · 10^−17^	0.9694	0.7269	0.8520	0.2357
1,000	0.01	3.6959 · 10^−18^ + 1.7917*j* · 10^−32^	4.5429 · 10^−18^	0.0724	0.1837	0.2268
1,000	0.1	3.2451 · 10^−32^ + 4.2776*j* · 10^−48^	2.5410 · 10^−32^	1.2004 · 10^−11^	0.0894	0.1934
10,000	0.001	5.2071 · 10^−18^ + 2.4675*j* · 10^−32^	6.0695 · 10^−18^	0.0659	0.1848	0.2432
10,000	0.01	5.7437 · 10^−30^ + 6.4925*j* · 10^−46^	5.4604 · 10^−30^	4.1244 · 10^−10^	0.0526	0.1873
10,000	0.1	3.9144 · 10^−32^ + 6.5162*j* · 10^−48^	2.9243 · 10^−32^	3.8058 · 10^−12^	3.2690 · 10^−10^	0.1245
20,000	0.001	7.2465 · 10^−28^ + 1.1227*j* · 10^−43^	6.3567 · 10^−28^	0.0057	0.1796	0.2289
20,000	0.01	5.3793 · 10^−30^ + 8.4984*j* · 10^−46^	5.4062 · 10^−30^	3.8469 · 10^−10^	0.0752	0.1859
20,000	0.1	3.0273 · 10^−32^ + 4.4578*j* · 10^−48^	3.3691 · 10^−32^	3.5718 · 10^−12^	7.1793 · 10^−11^	0.0556

Before we continue with the next set of experiments, it is important to mention that we performed the same experiments for finite domains bigger than the Boolean domain. The results showed that all functions had an exact solution, suggesting that the SP is not only able to learn exactly any *k*-ary Boolean function but may also have the ability to learn any *k*-ary *m*-valued function^6^. Such experiments can be found in the Supplementary Material and can be replicated using the GitHub repository.

Finally, all experiments in this part were conducted using only an AMD Ryzen 7 2700X Eight-Core Processor CPU.

We have shown that the signal perceptron and its variations always outperform the single hidden layer MLP when learning an entire Boolean function space. However, we would also like assess if this is also true when limited data is available and we are interested in learning a subset of functions. In other words, for the last set of experiments we were interested in measuring the generalization properties of the signal perceptrons when trained with practical datasets. As we mentioned above, we used to measure such generalization capabilities for the MNIST and FashionMNIST datasets. This two datasets consist of gray-scale images of 28 by 28 pixels, of handwritten numbers and clothes, respectively. All neural network architectures in this section where trained using the training set which consist on 60,000 examples and where tested on a test set of 10,000 examples for both MNIST and FashionMNIST.

For this set of experiments we evaluated performance of four different architectures. The first two will be FSPs using 128 and 512 signals, respectively. The last two will be a single hidden layer MLP of 512 nodes, and a MLP with two layers. Five hundred and twelve nodes in the first hidden layer and 512 in the second hidden layer. All MLPs used ReLu activation functions, and the weights of all architectures where initialized using the Kaiming initialization.

As in previous experiments, before training, we evaluated the spatial and computational complexity of the four architectures. For this reason, we measure the amount of learnable parameters, the average forward time in inference, and average backward time in training obtaining the results shown in Table 9.

**Table 9:** The spatial and computational complexity of different architectures for learning MNIST and FashionMNIST.

**Property**	**FSP-128**	**FSP-512**	**MLP_***h*1**_**	**MLP_***h*2**_**
Learnable parameters	**101,632**	406,528	407,050	669,706
Avg forward/inference time (ms)	**0.0889**	0.0894	0.1468	0.2142
Avg backward/backprop time (ms)	**0.8469**	1.0108	1.3031	1.1778

*128 signals FSP, 512 signals FSP, MLP of 1 hidden layer (512 nodes), and MLP of 2 hidden layers (512, 512). The results in bold letters indicate the model that has the best spatial and computational complexity*.

To ensure consistent results, the experiments were performed five times, and each time the weights were reinitialized. The averaged results of the five runs are shown in Table 10.

**Table 10:** Performance metrics by training different architectures on the MNIST and FashionMNIST datasets.

	**Performance metric**	**FSP-128**	**FSP-512**	**MLP_***h*1**_**	**MLP_***h*2**_**
**Mnist**					
	Avg training loss	0.0028	**0.0012**	0.2164	0.8635
	Avg test loss	0.0805	**0.0644**	0.2870	0.7757
	Avg accuracy	97.5%	**97.9%**	89.2%	67.9%
**FashionMnist**					
	Avg training loss	0.1342	**0.1323**	0.5977	0.7346
	Avg test loss	0.4034	**0.3902**	0.8041	1.2000
	Avg accuracy	86.0%	**86.3%**	68.5%	53.1%

*Used architectures: 128 signals FSP, 512 signals FSP, MLP of 1 hidden layer (512 nodes), and MLP of 2 hidden layers (512, 512). The results in bold letters indicate the model that achieved the best performance*.

As expected both FSP architectures outperformed the MLP variations. The most noticeable comparison is the inference and back-propagation runtimes between the FSP of 512 signals and the single hidden layer MLP. While the amount of parameters is almost the same, the MLP's inference time is at least 40% slower than that of the FSP and the training time is almost 30% slower.

For the training part, all the architectures were trained using the Adam optimizer with no dropout regularization and the cross-entropy loss function. The training analysis was made by running multiple times the training algorithm using both the MNIST and FahionMNIST datasets for 9 epochs with a learning rate of 0.001.

The results depicted in Table 10 are the averages of five runs of the same dataset. We can observe without any doubt that the model that performs the best is the FSP of 512 signals, scoring an average accuracy of 97.9% on the MNIST dataset and 86.3% on the FashionMNIST. As such being at least 10% more accurate than its MLP counterparts.

All experiments in this section were conducted on a Geforce GTX 1650 Super GPU.

## 6. Discussion

The main objective of the paper was to show the capability of the signal perceptron to learn all functions for any *k*-ary Boolean function space. Moreover, our experimental results imply that the general case of the SP may be expressive enough to learn any function, although this needs to be proven analytically. Regarding function approximation and generalization to unseen data, we showed that just a single signal perceptron outperforms the MLP architectures. Nonetheless, it is important to mention that this was achieved only with the FSP variation as there is an exponential blow-up in the number of parameters that define the SP and RSP in higher arities. Due to this, we will next discuss the limitations of the signal perceptron and provide a discussion for essential future work.

### 6.1. Limitations

One may reasonably develop concerns regarding the practical applicability of the SP and RSP when dealing with larger domains and arities. The source of such concern is the definition of the signal perceptron. As seen in Definition 6, the amount of learning parameters is equal to the number of signals. In consequence, exponentially many signals might be needed to guarantee that the whole functional space is learnable. However, as it has already been thoroughly discussed in previous sections, such a number is only necessary to analytically prove the expressiveness of the signal perceptron. Sections 2 and 4.4 showed that this limitation is also present in the MLP, which has not only an exponential blow-up but super-exponential regarding the number of parameters required to learn the whole functional space.

Still, as shown in Table 3, when learning particular functions not all signals from the signal perceptron will carry information. That is, the amplitudes of the signal are equal to zero. As such these signals are unnecessary and can be removed, which reduces the number of signals for certain functions. In addition, as discussed in Section 4.3 and shown in Section 5, when approximating functions we circumvented this apparent limitation by using the FSP, which outperformed the MLP in image classification tasks.

Moreover, this issue can also be potentially mitigated for the SP and the RSP. This could be achieved by defining Neural Networks of such mechanisms. As such we would utilize SPs or RSPs as units just like the perceptron are used as a unit in MLPs. Consider the following example over the MNIST dataset. A single Boolean signal perceptron with ten outputs will require 2^6,272^ parameters to learn the MNIST dataset. However, we could propose a Neural Network of Boolean signal perceptrons. As an example, the architecture could be defining the first layer with 28*28 perceptrons of 8 inputs for each pixel. Following a second layer with 88 perceptrons of 9 inputs, a third of 10 perceptrons of 9 inputs, and a final layer of 10 perceptrons with 10 inputs. By using this structure, the total number of required parameters becomes 720*256+512*88+512*10+1024*10 = 244736, which is less than the number of parameters used by the architectures in Section 5. This was shown in Table 9. Since our analysis was restricted to the capabilities of a single signal perceptron, such experiments are left for future work.

### 6.2. Comparison Against Other Prominent Neurons

As a continuation of Section 4.4, this section will consider a brief discussion between the signal perceptron variations and other types of neurons which attempt to solve the non-linear separability problem on Boolean function spaces with a single unit. This analysis is done to distinguish the contributions and advantages of the signal perceptron over other neurons in the literature. Having said that the analysis in this section is limited to **the learning of** **k-ary Boolean functions**, other comparisons for other discrete or continuous domains are out of scope.

The analysis will be focused on the following points.

- Structural Comparison. A brief analysis of the mathematical structure of these architectures, that will be used to articulate the main differences and similarities concerning the families of signal perceptrons. It is important to remark that no analysis over the function approximation capabilities will be conducted, given that our interest relies on learning the *k*-ary Boolean functions, which has been proved (and given that this is a finite family which is in particular PAC learnable Valiant, 1984). We could ask a different question: Is the family of *m*^*k*^-signal perceptrons is PAC learnable? This is certainly an interesting question that will be addressed in future work.
- Mathematical proof. One of the main contributions in this paper is the mathematical proof 4.2 of Theorem 1 in Section 4, which allowed us to claim that the signal perceptron and its variations can learn any *k*-ary Boolean function. If the discussed work doesn't provide such proof, then any claim that the mechanism solves the non-linear separability problem is likely only discussed empirically and thus is not directly comparable to the contributions of this paper.
- Spatial Complexity. Apart from the mathematical proof, we will look out for the topological analysis which will define an upper bound of the number of required parameters to learn any Boolean function. Without this upper bound, we will not be able to discern which architecture is preferable for the worst-case scenario.
- Learning algorithm. We will briefly describe the learning method used to train the structure. It was shown in Section 4 that, in the case of Boolean functions, we can learn the weights by solving a system of linear equations or by utilizing a back-propagation algorithm. A way to compare the time performance and computational complexity of the training method is by comparing these algorithms. Ideally, we would like the discussed neurons to be able to learn using back-propagation algorithms, since this is a way to assess the time performance and computational complexity, as it was done in Section 5.
- Experimental results. In this article we proved analytically that our mechanisms can learn any n-ary Boolean function. Nonetheless, form the experimental results of Section 5 and Appendix 1, we obtained the values of each learnable parameter of all binary and ternary Boolean functions as depicted in Tables 3, 4. It is important to remark that learned parameters obtained after training will yield a loss of zero. For this reason, comparisons of experimental results will not be measured in terms of accuracy and validation, and training error but in terms of how many functions the neurons were able to learn as shown in Table 5. This comparison will be done only for papers that perform experiments for learning Binary and ternary Boolean functions. An in-depth experimental analysis to compare training performance, computational complexity, and time complexity (as we did for the MLP in Section 5.1) is out of the scope of this paper.

A summarized version of the comparison can be found in Table 11, where we depict the key differences between the signal perceptron variations and the other neurons that try to solve the non-linear separability problem for Boolean functions. The first column of the table defines the training algorithm required for training the neurons. The second defines the upper bound of the number of learnable parameters required to learn binary, ternary, and any *k*-ary Boolean function. The third column depicts the experimental evaluation of the learning of binary and ternary Boolean functions, if it was conducted by the paper. For the single hidden layer MLP, Generalized Neuron and signal perceptron variations, results come from experiments conducted on Section 5 and Appendixes 1, 2. Finally, in the last column, we remark if the proposed neuron has been proven to be expressive enough to learn exactly any *k*-ary Boolean function.

**Table 11:** Summary table that compares the signal perceptron against the Rosenblatt perceptron, the 1-hidden-layer MLP, and some neurons in the machine learning literature that claim to solve the problem of learning non-linearly separable functions from Boolean function spaces.

		**Number of parameters (Upperbound)**			**Experimental evaluation**		**k-arity proof**	
**Neuron**	**Learning method**	**2-vars**	**3-vars**	**k-vars**	**2-vars**	**3-vars**	**Type**	**Result**
RP (Rosenblatt, 1958)	BP	3	4	(*k* + 1)	-	-	Math	Disproven
MLP (Baum, 1988)	BP	9	41	(*k* + 2)*m^*k*^ + 1	16/16	241/256	Math	Proven
*CVN*_1_ (Amin et al., 2008)	CBP	6	8	2(*k* + 1)	16/16	250/256	Experimental	Disproven
GN (Kulkarni and Venayagamoorthy, 2009)	BP/PSO	9	11	2(*k* + 1) + 3	14/16	114/256	Experimental	Disproven
DMN (Ritter and Urcid, 2003)	SLMP	8	48	2*k*m^*k*^	1/16	1/256	Math	Proven
SN (Maass and Schmitt, 1997)	-	-	-	-	-	-	Math	Disproven
SP (ours)	CBP/SLE	8	16	2*m^*k*^	16/16	256/256	Math	Proven
RSP (ours)	BP/SLE	4	8	*m* ^*k*^	16/16	256/256	Math	Proven
FSP (ours)	BP	8	24	k*m^*k*^	16/16	256/256	Math	Proven

*The table is divided into learning method, spatial complexity of each unit, experimental evaluation, and k-arity proof. Regarding the notation for the upper-bounds used to calculate the spatial complexity, the m term represents the domain size which for Boolean functions is m = 2, k is the arity of the input size of each of the neurons. The symbol − means that the information was missing, not discussed, or not proven by the paper*.

#### 6.2.1. Complex-Valued Neurons

The complex valued neuron CVN (Amin et al., 2008) is a function of the form:


(18) $$\begin{array}{l} o_{cvn1}=f_{act}\left(\left(\bar{\omega }\cdot e^{i\bar{x}}\right)+b\right)) \end{array}$$

where $\bar{\omega }\in {\mathbb{C}}^{n}$is a vector of complex weights, $\bar{x}\in {\mathbb{R}}^{n}$, is the input vector and *b* ∈ ℂ is a complex bias. The function *f*_*act*_ is a complex activation function that forces a mapping of the form ℂ → ℝ and is defined by:


(19) $$\begin{array}{l} \;f_{1act}\left(a+ib\right)=f_{1}\left(sig\left(a\right),sig\left(b\right)\right)=\sqrt{sig{\left(a\right)}^{2}+sig{\left(b\right)}^{2}}\\ \text{or:}\\ \;f_{2act}\left(a+ib\right)=f_{2}\left(sig\left(a\right),sig\left(b\right)\right)={\left(sig\left(a\right)-sig\left(b\right)\right)}^{2} \end{array}$$

where *a* + *ib* ∈ ℂ is the complex number, $sig\left(x\right)=\frac{1}{1+e^{-x}}$ is the sigmoid function, and *f*_1_, *f*_2_ are the quadratic functions $\sqrt{{\left(x_{1}\right)}^{2}+{\left(x_{2}\right)}^{2}},{\left(x_{1}-x_{2}\right)}^{2}$, respectively.

The process for calculating the output of the neuron can be summarized as follows. First the real input vector is encoded into a complex number using the non-linear function $\bar{z}=e^{i\bar{x}}$. Then, the resultant complex vector $\bar{z}$ is combined with the complex weights by the dot product $\bar{\omega }\cdot \bar{z}$ and added the bias *b*. Finally the resulting number is passed through one of the activation functions *f*_1_, *f*_2_ which force a mapping ℂ → ℝ From the Equations (18,19) and the forward pass description, we can conclude that the CVN is more closely related to Rosenblatt perceptron from Definition 3 than to the SP variations. That is:

**Proposition 2**. *The CVN is a particular variation of the Rosenblatt perceptron, where the partial operators* $\varphi_{1}\left(\bar{x}\right),\varphi_{2}\left(\bar{x}\right),...\varphi_{n}\left(\bar{x}\right)$ *use complex weights and the encoded input* $e^{i\bar{x}}$ *instead of real valued weights and the direct input. The join function* Ω *is defined by 2 non-linear functions in sequence rather than a single non-linear activation function*.

The CVN article does not provide mathematical proof that a single unit can solve the non-linear separability problem, but it provides an experimental analysis that shows that it is only able to solve the problem up to the binary Boolean functions, which makes it a less expressive model than the signal perceptron. Also, the paper does not give a topological analysis of how many CVNs will be required to guarantee that can learn the complete Boolean function spaces. The only analysis is for a single unit that defines the number of learnable parameters to be double as the parameters used in the Rosenblatt perceptron which is 2*k* + 2 where *k* is the arity of the Boolean function.

Regarding the training algorithm, the CVN article proposes an extension on gradient descent (CBP) to calculate the gradients for complex functions and weights. However, the learning algorithm is defined only to support the proposed activation functions and a particular loss function. On the other hand, the signal perceptron variations from Section 4.3 can be trained using normal back-propagation as they are already defined in the real domain. For the particular case of the signal perceptron, while it is required to compute the partial derivatives of complex numbers, such calculations are straightforward as shown in Algorithm 2. Calculating the gradients is also straight forward as we have a single non-linear function $e^{\frac{i\pi }{m-1}\left(\bar{\omega_{j}}\cdot \bar{x}\right)}$ whether the CVN requires 3 non-linear operations. One for the complex encoding $e^{i\bar{x}}$ and two for the activation functions *f*_1_, *f*_2_.

The experimental results do not seem to demonstrate that a single unit of a CVN can learn any *k*-ary Boolean function. This is observed when learning the ternary Boolean function space where the authors of the paper discusses that only 253/256 functions were learned using the activation function *f*_1*act*_ and 250/256 were learned using the activation function *f*_2*act*_. Unfortunately, those claims cannot be corroborated as no learned weights were provided in the results. The paper only provides empirical proof for the binary Boolean function space. They provide a table from the learned parameters for all binary Boolean functions, which shows that the CVN is only to solve the non-linear separability problem only for the Binary Boolean function space.

#### 6.2.2. Generalized Neuron

The Generalized Neuron (Kulkarni and Venayagamoorthy, 2009) is a function that can be formally defined as:


(20) $$\begin{array}{l} o_{gn}=\gamma f_{1}\left({\bar{w}}_{\sum }\cdot \bar{x}+b_{1}\right)+\left(1-\gamma \right)f_{2}\left(\left(\overset{k}{\underset{i=1}{\prod }}v_{i}x_{i}\right)b_{2}\right) \end{array}$$

where γ, *b*_1_, *b*_2_ ∈ ℝ are learnable parameters, $\bar{w},\bar{x}\in R^{k}$ is a weight and input vectors, respectively. Each variables *v*_*i*_, *x*_*i*_ are the particular values of the vectors $\bar{x},\bar{v}$ from the dimension *i* and *f*_1_, *f*_2_ are activation functions defined as:


(21) $$\begin{array}{l} f_{1}\left(x\right)=\frac{1}{1+e^{-\lambda_{1}x}}\\ f_{2}\left(x\right)=exp\left(-\lambda_{2}{\left(x\right)}^{2}\right) \end{array}$$

where λ_1_, λ_2_ ∈ ℝ are learnable parameters.

From Equations (20,21) we can observe that the generalized neuron can be defined as a composition of a Rossenblat neuron $f_{1}\left({\bar{w}}_{\sum }\cdot \bar{x}+b_{1}\right)$ and a neuron that uses a multiplication aggregation function. The final output is the aggregated result of both neurons outputs weighted by a γ parameter which defines the total contribution of each neuron.

To our best understanding, the paper doesn't provide a clear justification of the structure nor provides mathematical proof of the advantages of the GN neuron against the normal Rossenblatt perceptron. Indeed it is clear that since GN neuron should be more expressive than a single Rosenblatt perceptron as it can be thought of as a composition of 2 neurons. But such composition comes with the trade-off that the GN requires double the amount of parameters as the Rosenblatt perceptron. Thus, the authors of the paper define the spacial complexity of a single unit GN as 2(*k* + 1) + 3 where *k* is the arity of the function.

Regarding the claim that this neuron can solve the non-linearity separability problem on Boolean functions, the paper doesn't provide mathematical proof that a single unit of such mechanism is expressive enough to learn any *k*-ary Boolean function. From their experimental section, it is shown that it can learn the binary-xor function which can be used as a claim that can learn at least binary Boolean functions. As discussed for the CVN Section 6.2.1, the CVN was also able to learn binary Boolean functions but it failed to learn non-linear separable functions from the ternary Boolean function space when conducting their experiments. Thus, it remains unclear how the GN would work with ternary or higher arity functions, since as it is not obvious that experiments for the binary case generalize to higher arities.

The training method proposed in this article is particle swarm optimization (PSO) (Kennedy and Eberhart, 1995). The authors expressed the preference of this algorithm over stochastic gradient descent (SGD) since it was shown at that time that PSO outperformed it in some experimental tasks (Gudise and Venayagamoorthy, 2003). Since the publication of the article several optimizations and extensions have been defined for SGD reopening the question that PSO remains a more optimal algorithm than any BP extension.

The experimental section in Kulkarni and Venayagamoorthy (2009), briefly discusses the advantages of the GN against particular implementations of the single hidden layer MLP to learn non-linear functions. The first set of experiments compares both structures by approximating single input continuous non-linear functions defined over a time domain. For the Boolean domain, the only experiment conducted was to learn the binary XOR function. As stated before, this single experiment is not sufficient to support any claim that the GN is expressive enough to learn any arbitrary *k*-ary Boolean function. In Appendix 2 we train multiple times the GN to learn all the binary and ternary Boolean functions using SGD. Our experimental results show that for the ternary Boolean function space, the GN could learn at most 114 the 256 functions. This result provides empirical proof of our assumption that a single unit of GN is not expressive enough to learn any *k*-ary Boolean function.

#### 6.2.3. Dendrite Morphological Neurons

A Dentrite Morphological Neuron DNM (Maass and Schmitt, 1997) is defined as a function that has the following form:


(22) $$\begin{array}{l} o_{dmn}=f_{act}\left(\overset{N}{\underset{n=1}{⋀}}\left[p_{n}\overset{k}{\underset{i=1}{⋀}}\underset{l\in \left\{0,1\right\}}{⋀}{\left(-1\right)}^{\left(1-l\right)}\left(x_{k}+w_{kn}^{l}\right)\right]\right) \end{array}$$

Where *x* ∈ ℝ^*k*^, *p*_*n*_ ∈ {1, −1}, $w_{kn}^{l}\in \mathbb{R}$, are the weights, and *N* is the number of dendrites, and the activation function, *f*_*act*_, is a step function.

In their paper, they prove a theorem that says that in a Euclidean space ℝ^*n*^, for any compact subset *A* ⊆ ℝ^*n*^ and ϵ > 0 there exists a single layer DMN *o*_*dmn*_(*x*) = *f*_*act*_(*g*(*x*)) that will divide the space in two regions, $C_{0}=f^{-1}\left(0\right),C_{1}=f^{-1}\left(1\right)$ such that for every *x* ∈ ℝ^*n*^, *d*(*x, X*) > ϵ^7^, if and only if *x* ∈ *C*_0_. It is important to note that this theorem only proves the existence of such function, but doesn't say anything about its form, in particular, about the required number of dendrites that are needed.

How could we use a DMN to learn a Boolean function *h*:2^*n*^ → 2? One way would be to take the compact set *h*^−1^(1) ⊆ ℝ^*n*^ and use the previous theorem for any ϵ > 0. As such, there exists a DMN *f* such that for all *x* ∈ 2^*n*^, *f*(*x*) = *h*(*x*). The function is characterized by the weights $\left\{w_{ik}^{l}\right\}$, therefore we need to learn 2*kN* parameters. Unfortunately, the theorem doesn't provide the number of dendrites required to learn any Boolean *k*-ary function. Nonetheless, in the conclusion the authors make the following claim:

In comparison to hidden layer neurons which generally use sigmoidal activation functions, dendrites have no activation functions. They only compute the basic logic functions of AND, OR, and NOT. (Ritter and Urcid, 2003)

This means that if we express the function *h* as a propositional formula with *k* variables in Conjunctive Normal Form (CNF), then each dendrite is computing a clause with the function


$$\tau_{n}\left(x\right)=p_{n}\overset{k}{\underset{i=1}{⋀}}\underset{l\in \left\{0,1\right\}}{⋀}{\left(-1\right)}^{\left(1-l\right)}\left(x_{i}+w_{in}^{l}\right)$$

and the infimum of all these values is the conjunction of all the clauses. This gives us a maximum number of dendrites needed, 2^*k*^ (Russell and Norvig, 2010), which is the same as the maximum of clauses needed. Therefore, an upper bound for the number of parameters that are needed to learn a function *h* : 2^*k*^ → 2 with a DMN is 2*Nk* = 2^*k*+1^*k*, which is way higher than for the SP, whose upper bound is 2^*k*^.

The experimental evaluation for Boolean functions given in this paper consists only on the binary and ternary XOR. Rather than using a back-propagation method, the authors of the paper propose a learning method called SLMP training. This is a supervised learning method, in which the training set is *T* = {(*x*^ξ^, *c*^ξ^) : ξ ∈ {1, ..., *m*}}, where *c*^ξ^ ∈ {0, 1} and $x^{\xi }\in C_{j}$ if *c*^ξ^ = *j*. The algorithm first calculates a DMN and if it fails it adds another dendrite. If the number of dendrites is *N* (which means that we are in the *N*-th iteration) then to define the function of the *n*-th dendrite


$$\tau_{n}\left(x^{\xi }\right)=p_{n}\overset{k}{\underset{i=1}{⋀}}\overset{k}{\underset{i=1}{⋀}}\underset{l\in \left\{0,1\right\}}{⋀}r_{in}^{l}\left(x_{i}^{\xi }+w_{in}^{l}\right)$$

they define the value of the coefficients as,


$$w_{in}^{1}=-\underset{x^{\xi }\in C_{1}}{⋀}x_{i}^{\xi }\;w_{in}^{1}=-\underset{x^{\xi }\in C_{1}}{⋁}x_{i}^{\xi }$$

$$p_{n}={\left(-1\right)}^{sgn(n-1)8}\;r_{in}^{l}={\left(-1\right)}^{1-l}$$

Given this, and a threshold τ that depends on the degree of accuracy ϵ, the total response of the whole neuron is given by the function


$$\tau \left(x^{\xi }\right)=\overset{k}{\underset{n=1}{⋀}}\tau_{j}\left(x^{\xi }\right)$$

Then they use the Heaviside unit step hard limiter as activation function *f*^9^ and check if


$$f\left(\tau \left(x^{\xi }\right)\right)=c^{\xi }$$

If this is false i.e., the equality is not satisfied, then they add another dendrite and repeat the procedure.

#### 6.2.4. Spiking Neuron

A spiking neuron of type A (SN) (Maass, 1997) is a function of the form:


(23) $$\begin{array}{l} o_{sn}(t)=f_{act}(\sum_{i=1}^{k}h_{i}(t-t_{i})) \end{array}$$

where each *h*_*i*_ is defined by a weight *w*_*i*_ ∈ ℝ and a delay $d_{i}\in {\mathbb{R}}^{<0}$ as


(24) $$\begin{array}{l} h_{i}(t)=\{\begin{array}{ll} 0 & \text{for }t<d_{i}\text{ or }t\geq d_{i}+1,\\ w_{i} & \text{for }d_{i}\leq t<d_{i}+1 \end{array} \end{array}$$

Each *h*_*i*_ a pulse that starts at time *t*_*i*_ with an intensity of *w*_*i*_ and remains for a time *d*_*i*_. The idea is that the neuron will fire when the sum of the pulses reaches a certain threshold determined by the activation function. Therefore, if *N* is the number of pulses of the SN, then the number of parameters needed to characterize a SN is 2*N* + 1, one for the threshold and a weight and delay per pulse.

Given that the SN neurons require weights and delay parameters, their computational complexity will be larger than Rosenblatt's percepetron (which only requires the weights). A more in-depth analysis of the advantages of the spiking neuron against the Rosenblatt perceptron can be found at (Schmitt, 1998).

In the Theorem 2.1 (e) of their paper, they state that if *k* ≥ 2 then there are Boolean functions that can't be computed by a spiking neuron with binary coding of the inputs. They also provide an upper bound of the amount of Boolean functions that can be computed by a SN, which is 2^*n*^2^+*O*(*nlog*(*n*))^. Therefore, the spiking neuron is not as expressive as the signal perceptron, given that it doesn't learn the space of Boolean functions.

Finally, it is important to mention that no training algorithm nor experimental section were provided in both articles (Maass, 1997; Maass and Schmitt, 1997), as their main concern is on the definition of the SN mechanism and its learning complexity.

### 6.3. Future Work

A promising direction for future work is to provide an analytic proof of Proposition 1 for the general case of the signal perceptron as shown in Definition 6. We conducted some preliminary experiments for different arities and *n*-valued function spaces and found that the matrices generated by the signal perceptron were all invertible matrices^10^. While these results are promising, the formal proof is still required to state that the general signal perceptron can indeed learn any function from any finite function space. Furthermore, as shown in the proof4.2, the *k*-ary Boolean signal perceptron generates Walsh Matrices. An interesting question that arises from this is whether the matrices formed by the general definition of the signal perceptron are also a generalization of Walsh matrices or Hadamard matrices. Future work could look into answering this open question.

We haven't formally investigated the learning complexity of the signal perceptron variations. One way to do this, which will be addressed in future work, is by calculating the VC Dimension (Vapnik, 1995) of the family of *m*^*k*^-SP. One way of seeing the VC Dimension of a family of binary classifiers H is as the cardinality of the largest set *C* such that any subset of *C* can be classified with a classifier in H–In the literature this denoted by saying that *C* is *shattered* by H. The VC dimension is an important quantity in ML because it characterizes PAC learnability^11^, and provides lower and upper bounds for the sample complexity of the family of functions (Shalev-Shwartz and Ben-David, 2014). In this paper we have proved that the family of 2^*k*^-SP has VC dimension larger than 2^*k*^, this is a consequence that it can learn any *k*-ary Boolean function^12^. To finish the proof that the VC dimension is finite, we would have to prove that there exists a finite set *C* that can't be shattered by the family of 2^*k*^-SP. Given that the parameters of the SP are determined by the amplitudes of the signals and not the frequencies, there are good chances that this can happen and therefore, it is PAC learnable and we can obtain bounds for the sample complexity and give a finer comparison with the other neurons.

As mentioned previously in Section 6.1, there exists the potential to create neural networks of signal perceptrons. Moreover, all the experiments conducted in Section 5 used a single signal perceptron unit or one of its variations. This leaves the domain of multilayer signal perceptrons and their capabilities completely unexplored.

Last but not least, this new mechanism could be used in combination with–or to create new variations of–existing mechanisms and architectures used in deep learning such as Convolutional Neural Networks (Krizhevsky et al., 2012), Generative Adversarial Networks (Goodfellow et al., 2014), VariationalAutoencoders (Doersch, 2016), and Transformers (Vaswani et al., 2017). As a consequence, the capabilities of such combinations and modifications are yet to be explored.

The scope of the present paper is limited to Boolean functions and categorical datasets. To effectively confirm a broader scope of the practical applications and performance of the SP against state-of-the-art DL models, we would need to further explore and analyze its performance on different types of datasets.

Finally, the use of signal perceptrons could potentially open a whole branch of deep learning analysis. That is because the whole architecture of the signal perceptron defines a complex signal or a real signal. The use of signal analysis over such architectures may lay down the basis for more interpretable deep learning mechanisms.

### 6.4. Conclusions

We have defined the signal perceptron, a new mathematical mechanism for learning functions. We proved that such a mechanism can learn any function from any *k*-ary Boolean function space with just a single unit. We also defined two variations of the mechanism that overcome some of its limitations. Furthermore, we showed that this novel mechanism requires fewer parameters for learning a whole function space when compared to the single hidden layer MLP. Our experiments showed that the capabilities of signal perceptrons are not restricted to just learning *k*-ary Boolean function spaces but can also be used as function approximation methods. In such a scenario, they outperformed the MLP when trained in image classification tasks. Finally, only the DMN and Signal perceptron variations were the only structures expressive enough to learn any arbitrary *k*-ary Boolean function with a single unit. This results from the comparison of Section 6.2 with other neurons that attempt to solve the non-linear function learning problem for the Boolean domain. We also showed that in terms of spatial complexity all signal perceptron variations require fewer parameters for the worst-case scenario than the DMN and SN which makes it a more efficient structure for learning Boolean functions.

## Data Availability Statement

Source code for the algorithms and data used for the experiments in this paper can be found in the following public GitHub repository: https://github.com/miguelamendez/SignalPerceptron. Further inquiries can be directed to the corresponding author.

## Author Contributions

Idea was conceived, implemented, and written by M-AM. EB provided help with the implementation, writing, and proofs. VB and R-MK supervised the work and writing of the paper. All authors have made direct and intellectual contributions to this work and have approved it for publication.

## Funding

This work was partly supported by the EPSRC grant Toward Explainable and Statistical AI: A Symbolic Approach, and partly by a grant from the UKRI Strategic Priorities Fund to the UKRI Research Node on Trustworthy Autonomous Systems Governance and Regulation (EP/V026607/1, 2020-2024). VB is also supported by a Royal Society University Research Fellowship. M-AL is supported by Consejo Nacional de Ciencia y Tecnología CONACYT.

## Conflict of Interest

The authors declare that the research was conducted in the absence of any commercial or financial relationships that could be construed as a potential conflict of interest.

## Publisher's Note

All claims expressed in this article are solely those of the authors and do not necessarily represent those of their affiliated organizations, or those of the publisher, the editors and the reviewers. Any product that may be evaluated in this article, or claim that may be made by its manufacturer, is not guaranteed or endorsed by the publisher.

## Footnotes

^1^In Section 4.3 we provide a variation of such mechanism that can work with continuous domains.

^2^For analytic signals the α parameter is a complex number that defines the amplitude and the phase of a signal. For the case of the real signals the α parameter is a real number representing amplitudes and half phase shift.

^3^Both Implementations can be found in the following GitHub repository: https://github.com/miguelamendez/SignalPerceptron.

^4^Results of similar experiments are provided in the Appendix 1 for ternary Boolean functions.

^5^Such experiments can be found in the GitHub repository https://github.com/miguelamendez/SignalPerceptron.

^6^This must be analytically proved though.

^7^*d*(*x, a*) is the distance of the point *x* to the set *A* i.e., *d*(*x, A*) = *inf*{*d*(*x, y*)|*y* ∈ *A*}.

^9^*f*(*x*) = 1 if *x* ≥ 0, and *f*(*x*) = 0 if *x* < 0.

^10^Experiments can be found in the GitHub repository.

^11^A family of functions has finite VC dimension if and only if it is PAC learnable.

^12^The set of *k*-ary Boolean functions is isomorphic to the subsets of the set 2^*k*^.

## References

[B1] Amin M. F. Islam M. M. Murase K. (2008). Single-layered complex-valued neural networks and their ensembles for real-valued classification problems, in 2008 IEEE International Joint Conference on Neural Networks (IEEE World Congress on Computational Intelligence) (Hong Kong: IEEE), 2500–2506.

[B2] Banda P. Teuscher C. (2014). Learning two-input linear and nonlinear analog functions with a simple chemical system, in International Conference on Unconventional Computation and Natural Computation (Ontario: Springer), 14–26.

[B3] Baum E. B. (1988). On the capabilities of multilayer perceptrons. J. Complex 4, 193–215. 10.1016/0885-064X(88)90020-9

[B4] Bebis G. Georgiopoulos M. (1994). Feed-forward neural networks. IEEE Potentials 13, 27–31. 10.1109/45.329294

[B5] Blount D. Banda P. Teuscher C. Stefanovic D. (2017). Feedforward chemical neural network: an in silico chemical system that learns xor. Artif. Life 23, 295–317. 10.1162/ARTL_a_00233 28786723

[B6] Bottou L. Curtis F. E. Nocedal J. (2018). Optimization methods for large-scale machine learning. SIAM Rev. 60, 223–311. 10.1137/16M1080173

[B7] Cazé R. D. Humphries M. Gutkin B. (2013). Passive dendrites enable single neurons to compute linearly non-separable functions. PLoS Comput. Biol. 9, e1002867. 10.1371/journal.pcbi.1002867 23468600 PMC3585427

[B8] Cheolwoo Y. Daesik H. (1998). Nonlinear blind equalization schemes using complex-valued multilayer feedforward neural networks. IEEE Trans. Neural Netw. 9, 1442–1455. 10.1109/72.728394 18255822

[B9] Clarke T. L. (1990). Generalization of neural networks to the complex plane, in 1990 IJCNN International Joint Conference on Neural Networks, 435–440.

[B10] Cybenko G. (1989). Approximation by superpositions of a sigmoidal function. Math. Control Signals Syst. 2, 303–314. 10.1007/BF02551274

[B11] Devlin J. Chang M.-W. Lee K. Toutanova K. (2019). BERT: pre-training of deep bidirectional transformers for language understanding, in Proceedings of the 2019 Conference of the North American Chapter of the Association for Computational Linguistics: Human Language Technologies, Volume 1 (Long and Short Papers) (Minneapolis, MN: Association for Computational Linguistics), 4171–4186.

[B12] Doersch C. (2016). Tutorial on variational autoencoders. arxiv:1606.05908. 10.48550/arXiv.1606.05908

[B13] Ferrag M. A. Maglaras L. Moschoyiannis S. Janicke H. (2020). Deep learning for cyber security intrusion detection: approaches, datasets, and comparative study. J. Inform. Security Appl. 50, 102419. 10.1016/j.jisa.2019.10241934640752

[B14] Goodfellow I. Pouget-Abadie J. Mirza M. Xu B. Warde-Farley D. Ozair S.. (2014). Generative adversarial nets, in Advances in Neural Information Processing Systems, Vol. 27, eds Ghahramani Z. Welling M. Cortes C. Lawrence N. Weinberger K. Q. (Montreal, QC: Curran Associates, Inc.).

[B15] Gruzling N. (2007). Linear separability of the vertices of an n-dimensional hypercube. UNBC. 10.24124/2007/bpgub464

[B16] Gudise V. Venayagamoorthy G. (2003). Comparison of particle swarm optimization and backpropagation as training algorithms for neural networks, in Proceedings of the 2003 IEEE Swarm Intelligence Symposium. SIS'03 (Cat. No.03EX706) (Indianapolis, IN: IEEE), 110–117.

[B17] He K. Zhang X. Ren S. Sun J. (2016). Deep residual learning for image recognition, in 2016 IEEE Conference on Computer Vision and Pattern Recognition (CVPR) (Las Vegas, NV: IEEE), 770–778.

[B18] Hertz J. A. Krogh A. S. Palmer R. G. (1991). Introduction To The Theory of Neural Computation. Reading. MA: Addison-Wesley.

[B19] Huang G.-B. (2003). Learning capability and storage capacity of two-hidden-layer feedforward networks. Trans. Neur. Netw. 14, 274–281. 10.1109/TNN.2003.809401 18238011

[B20] Huang G.-B. Babri H. (1997). General approximation theorem on feedforward networks, in Proceedings of ICICS, 1997 International Conference on Information, Communications and Signal Processing. Theme: Trends in Information Systems Engineering and Wireless Multimedia Communications (Singapor), 698–702. 10.1109/ICICS.1997.652067

[B21] Huh D. Sejnowski T. J. (2018). Gradient descent for spiking neural networks, in Advances in Neural Information Processing Systems, Vol 31, eds Bengio S. Wallach H. Larochelle H. Grauman K. Cesa-Bianchi N. Garnett R. (Curran Associates, Inc.). Available online at: https://proceedings.neurips.cc/paper/2018/file/185e65bc40581880c4f2c82958de8cfe-Paper.pdf

[B22] Kanjilal P. P. (1995). Adaptive prediction and predictive control, in Control, Robotics andamp; Sensors. Institution of Engineering and Technology.

[B23] Kennedy J. Eberhart R. (1995). Particle swarm optimization, in Proceedings of ICNN'95-International Conference on Neural Networks, Vol. 4 (Perth, WA), 1942–1948.

[B24] Kim T. Adali T. (2002). Fully complex multi-layer perceptron network for nonlinear signal processing. VLSI Signal Process. 32, 29–43. 10.1023/A:101635921696133632237

[B25] Krizhevsky A. Sutskever I. Hinton G. E. (2012). Imagenet classification with deep convolutional neural networks, in Advances in Neural Information Processing Systems, Vol. 25, eds Pereira F. Burges C. J. C. Bottou L. Weinberger K. Q. (Lake Tahoe: Curran Associates, Inc.).

[B26] Kulkarni R. V. Venayagamoorthy G. K. (2009). Generalized neuron: Feedforward and recurrent architectures. Neural Netw. 22, 1011–1017. 10.1016/j.neunet.2009.07.027 19660907

[B27] Kůrková V. (1992). Kolmogorov's theorem and multilayer neural networks. Neural Netw. 5, 501–506. 10.1016/0893-6080(92)90012-8

[B28] LeCun Y. Cortes C. (2010). MNIST handwritten digit database. IEEE Signal Process. Mag. 29, 141–142. Available online at: https://www.bibsonomy.org/bibtex/2935bad99fa1f65e03c25b315aa3c1032/mhwombat

[B29] Maass W. (1997). Networks of spiking neurons: The third generation of neural network models. Neural Netw. 10, 1659–1671. 10.1016/S0893-6080(97)00011-7

[B30] Maass W. Schmitt M. (1997). On the complexity of learning for a spiking neuron (extended abstract), in Proceedings of the Tenth Annual Conference on Computational Learning Theory, COLT '97 (New York, NY: Association for Computing Machinery), 54–61.

[B31] Minsky M. (1969). Perceptrons: An Introduction to Computational Geometry. Cambridge, MA: The MIT Press.

[B32] Mondal R. Mukherjee S. S. Santra S. Chanda B. (2020). Morphological network: how far can we go with morphological neurons? arXiv:1901.00109. 10.48550/arXiv.1901.00109

[B33] Nitta T. (2003). Solving the xor problem and the detection of symmetry using a single complex-valued neuron. Neural Netw. 16, 1101–1105. 10.1016/S0893-6080(03)00168-0 13678617

[B34] Pisarev A. Busygin A. Udovichenko S. Y. Maevsky O. (2020). A biomorphic neuroprocessor based on a composite memristor-diode crossbar. Microelectron. J. 102, 104827. 10.1016/j.mejo.2020.104827

[B35] Ritter G. Urcid G. (2003). Lattice algebra approach to single-neuron computation. IEEE Trans. Neural Netw. 14, 282–295. 10.1109/TNN.2003.809427 18238012

[B36] Rosenblatt F. (1958). The perceptron: a probabilistic model for information storage and organization in the brain. Psychol. Rev. 65, 386–408. 10.1037/h0042519 13602029

[B37] Russell S. Norvig P. (2010). Artificial intelligence: a modern approach, in Prentice Hall series in artificial intelligence (Upper Saddle River, NJ: Pearson).

[B38] Schmitt M. (1998). On computing boolean functions by a spiking neuron. Ann. Math. Artif. Intell. 24, 181–191. 10.1023/A:101895330018523468600

[B39] Shalev-Shwartz S. Ben-David S. (2014). Understanding Machine Learning - From Theory to Algorithms. New York, NY: Cambridge University Press.

[B40] Shannon C. E. (1938). A symbolic analysis of relay and switching circuits. Trans. Inst. Electr. Eng. 57, 713–723. 10.1109/T-AIEE.1938.5057767

[B41] Silver D. Huang A. Maddison C. J. Guez A. Sifre L. van den Driessche G.. (2016). Mastering the game of Go with deep neural networks and tree search. Nature 529, 484–489. 10.1038/nature16961 26819042

[B42] Simonyan K. Zisserman A. (2015). Very deep convolutional networks for large-scale image recognition. arXiv arXiv:1409.1556. 10.48550/arXiv.1409.1556

[B43] Smith J. (2010). Mathematics of the Discrete Fourier Transform (DFT), 2nd edn. BookSurge. Available online at: https://www.bibsonomy.org/bibtex/2d529258218c89de526008d726d90d433/ytyoun

[B44] Srivastava N. Hinton G. Krizhevsky A. Sutskever I. Salakhutdinov R. (2014). Dropout: a simple way to prevent neural networks from overfitting. J. Mach. Learn. Res. 15, 1929–1958. 10.5555/2627435.2670313

[B45] Stathakis D. (2009). How many hidden layers and nodes? Int. J. Remote Sens. 30, 2133–2147. 10.1080/01431160802549278

[B46] Tavanaei A. Ghodrati M. Kheradpisheh S. R. Masquelier T. Maida A. (2019). Deep learning in spiking neural networks. Neural Netw. 111, 47–63. 10.1016/j.neunet.2018.12.002 30682710

[B47] Valiant L. G. (1984). A theory of the learnable, in Proceedings of the Sixteenth Annual ACM Symposium on Theory of Computing, STOC '84 (New York, NY: Association for Computing Machinery), 436–445.

[B48] Vapnik V. N. (1995). The Nature of Statistical Learning Theory. New York, NY: Springer-Verlag New York, Inc.

[B49] Vaswani A. Shazeer N. Parmar N. Uszkoreit J. Jones L. Gomez A. N.. (2017). Attention is all you need, in Advances in Neural Information Processing Systems, Vol. 30, eds Guyon I. Luxburg U. V. Bengio S. Wallach H. Fergus R. Vishwanathan S. Garnett R. (Long Beach, CA: Curran Associates, Inc.).

[B50] Wilson E. Tufts D. W. (1994). Multilayer perceptron design algorithm, in Proceedings of IEEE Workshop on Neural Networks for Signal Processing (Ermioni: IEEE), 61–68.

[B51] Xiao H. Rasul K. Vollgraf R. (2017). Fashion-mnist: a novel image dataset for benchmarking machine learning algorithms. arxiv:1708.07747. 10.48550/arXiv.1708.07747

[B52] Zhang X. (2013). Neural Networks in Optimization. Nonconvex Optimization and Its Applications. Boston, MA: Springer U.S.

