# Supplementary material for: Signal Perceptron: On the Identifiability of Boolean Function Spaces and Beyond
Source: Front Artif Intell. 2022 Jun 2;5:770254. doi: 10.3389/frai.2022.770254 (PMC9203047; doi:10.3389/frai.2022.770254)
Supplement: Supplementary file 1 [file Data_Sheet_1.zip › SignalPerceptron_SupplementaryMatterial/signalPerceptron_Supplementary_Material.pdf]

Supplementary Material

1 APPENDIX 1

Experimental results obtained when learning all functions from the the ternary Boolean Function Space. The training method used in this section was back-propagation using an Adam optimizer with no regularization and a fixed learning rate of .1. All the architectures were trained using batch gradient descent with no dropout regularization (Srivastava et al., 2014) and the mean squared error (MSE) loss function. The all the results in this Appendix are the average of running the experiments for 5 consecutive times unless specified otherwise.

Table S1. Average final loss for different implementations of the signal perceptron and single-layer MLP for learning the 256 functions of the ternary Boolean function space.

Epochs	SP NumPy	RSP NumPy	RSP PyTorch	FSP PyTorch	MLP$_{h1}$ PyTorch
100	$0.0 + 0j$	0.0	0.0	0.0001	0.0103
1000	$0.0 + 0j$	0.0	0.0	0.0	0.0077
2000	$0.0 + 0j$	0.0	0.0	0.0	0.0039

Table S2. Amount of ternary Boolean functions learned by the different implementations of the signal perceptron variations and MLP with one hidden layer. We define that a function is learned when the final loss is less than .001. The number of Boolean functions displayed in this table is the maximum number learned after conducting the experiments five times

Epochs	SP NumPy	RSP NumPy	RSP PyTorch	FSP PyTorch	MLP$_{h1}$ PyTorch
100	256	256	256	253	140
1000	256	256	256	256	234
2000	256	256	256	256	241

Table S3: Table of all parameters required by the SP and RSP to define every ternary Boolean function. These were obtained by running an implementation of Algorithms **??** and **??**.

**Function**	$\alpha_1, \alpha_2, \alpha_3, \alpha_4, \alpha_5, \alpha_6, \alpha_7, \alpha_8$	**Function**	$\alpha_1, \alpha_2, \alpha_3, \alpha_4, \alpha_5, \alpha_6, \alpha_7, \alpha_8$
0 0 0 0 0 0 0 0	0 0 0 0 0 0 0 0	0 0 0 0 0 0 0 1	0.125 -0.125 -0.125 0.125 -0.125 0.125 0.125 -0.125
1 0 0 0 0 0 0 0	0.125 0.125 0.125 0.125 0.125 0.125 0.125 0.125	1 0 0 0 0 0 0 1	0.25 0 0 0.25 0 0.25 0.25 0
0 1 0 0 0 0 0 0	0.125 -0.125 0.125 -0.125 0.125 -0.125 0.125 -0.125	0 1 0 0 0 0 0 1	0.25 -0.25 0 0 0 0 0.25 -0.25
1 1 0 0 0 0 0 0	0.25 0 0.25 0 0.25 0 0.25 0	1 1 0 0 0 0 0 1	0.375 -0.125 0.125 0.125 0.125 0.125 0.375 -0.125
0 0 1 0 0 0 0 0	0.125 0.125 -0.125 -0.125 0.125 0.125 -0.125 -0.125	0 0 1 0 0 0 0 1	0.25 0 -0.25 0 0 0.25 0 -0.25
1 0 1 0 0 0 0 0	0.25 0.25 0 0 0.25 0.25 0 0	1 0 1 0 0 0 0 1	0.375 0.125 -0.125 0.125 0.125 0.375 0.125 -0.125
0 1 1 0 0 0 0 0	0.25 0 0 -0.25 0.25 0 0 -0.25	0 1 1 0 0 0 0 1	0.375 -0.125 -0.125 -0.125 0.125 0.125 0.125 -0.375
1 1 1 0 0 0 0 0	0.375 0.125 0.125 -0.125 0.375 0.125 0.125 -0.125	1 1 1 0 0 0 0 1	0.5 0 0 0 0.25 0.25 0.25 -0.25
0 0 0 1 0 0 0 0	0.125 -0.125 -0.125 0.125 0.125 -0.125 -0.125 0.125	0 0 0 1 0 0 0 1	0.25 -0.25 -0.25 0.25 0 0 0 0
1 0 0 1 0 0 0 0	0.25 0 0 0.25 0.25 0 0 0.25	1 0 0 1 0 0 0 1	0.375 -0.125 -0.125 0.375 0.125 0.125 0.125 0.125
0 1 0 1 0 0 0 0	0.25 -0.25 0 0 0.25 -0.25 0 0	0 1 0 1 0 0 0 1	0.375 -0.375 -0.125 0.125 0.125 -0.125 0.125 -0.125
1 1 0 1 0 0 0 0	0.375 -0.125 0.125 0.125 0.375 -0.125 0.125 0.125	1 1 0 1 0 0 0 1	0.5 -0.25 0 0.25 0.25 0 0.25 0
0 0 1 1 0 0 0 0	0.25 0 -0.25 0 0.25 0 -0.25 0	0 0 1 1 0 0 0 1	0.375 -0.125 -0.375 0.125 0.125 0.125 -0.125 -0.125
1 0 1 1 0 0 0 0	0.375 0.125 -0.125 0.125 0.375 0.125 -0.125 0.125	1 0 1 1 0 0 0 1	0.5 0 -0.25 0.25 0.25 0.25 0 0
0 1 1 1 0 0 0 0	0.375 -0.125 -0.125 -0.125 0.375 -0.125 -0.125 -0.125	0 1 1 1 0 0 0 1	0.5 -0.25 -0.25 0 0.25 0 0 -0.25
1 1 1 1 0 0 0 0	0.5 0 0 0 0.5 0 0 0	1 1 1 1 0 0 0 1	0.625 -0.125 -0.125 0.125 0.375 0.125 0.125 -0.125
0 0 0 0 1 0 0 0	0.125 0.125 0.125 0.125 -0.125 -0.125 -0.125 -0.125	0 0 0 0 1 0 0 1	0.25 0 0 0.25 -0.25 0 0 -0.25
1 0 0 0 1 0 0 0	0.25 0.25 0.25 0.25 0 0 0 0	1 0 0 0 1 0 0 1	0.375 0.125 0.125 0.375 -0.125 0.125 0.125 -0.125
0 1 0 0 1 0 0 0	0.25 0 0.25 0 0 -0.25 0 -0.25	0 1 0 0 1 0 0 1	0.375 -0.125 0.125 0.125 -0.125 -0.125 0.125 -0.375
1 1 0 0 1 0 0 0	0.375 0.125 0.375 0.125 0.125 -0.125 0.125 -0.125	1 1 0 0 1 0 0 1	0.5 0 0.25 0.25 0 0 0.25 -0.25
0 0 1 0 1 0 0 0	0.25 0.25 0 0 0 0 -0.25 -0.25	0 0 1 0 1 0 0 1	0.375 0.125 -0.125 0.125 -0.125 0.125 -0.125 -0.375
1 0 1 0 1 0 0 0	0.375 0.375 0.125 0.125 0.125 0.125 -0.125 -0.125	1 0 1 0 1 0 0 1	0.5 0.25 0 0.25 0 0.25 0 -0.25
0 1 1 0 1 0 0 0	0.375 0.125 0.125 -0.125 0.125 -0.125 -0.125 -0.375	0 1 1 0 1 0 0 1	0.5 0 0 0 0 0 0 -0.5
1 1 1 0 1 0 0 0	0.5 0.25 0.25 0 0.25 0 0 -0.25	1 1 1 0 1 0 0 1	0.625 0.125 0.125 0.125 0.125 0.125 0.125 -0.375
0 0 0 1 1 0 0 0	0.25 0 0 0.25 0 -0.25 -0.25 0	0 0 0 1 1 0 0 1	0.375 -0.125 -0.125 0.375 -0.125 -0.125 -0.125 -0.125
1 0 0 1 1 0 0 0	0.375 0.125 0.125 0.375 0.125 -0.125 -0.125 0.125	1 0 0 1 1 0 0 1	0.5 0 0 0.5 0 0 0 0
0 1 0 1 1 0 0 0	0.375 -0.125 0.125 0.125 0.125 -0.375 -0.125 -0.125	0 1 0 1 1 0 0 1	0.5 -0.25 0 0.25 0 -0.25 0 -0.25

Continued on next page

Table S3 – continued from previous page

Function	$\alpha_1, \alpha_2, \alpha_3, \alpha_4, \alpha_5, \alpha_6, \alpha_7, \alpha_8$	Function	$\alpha_1, \alpha_2, \alpha_3, \alpha_4, \alpha_5, \alpha_6, \alpha_7, \alpha_8$
1 1 0 1 1 0 0 0	0.5 0 0.25 0.25 0.25 -0.25 0 0	1 1 0 1 1 0 0 1	0.625 -0.125 0.125 0.375 0.125 -0.125 0.125 -0.125
0 0 1 1 1 0 0 0	0.375 0.125 -0.125 0.125 0.125 -0.125 -0.375 -0.125	0 0 1 1 1 0 0 1	0.5 0 -0.25 0.25 0 0 -0.25 -0.25
1 0 1 1 1 0 0 0	0.5 0.25 0 0.25 0.25 0 -0.25 0	1 0 1 1 1 0 0 1	0.625 0.125 -0.125 0.375 0.125 0.125 -0.125 -0.125
0 1 1 1 1 0 0 0	0.5 0 0 0 0.25 -0.25 -0.25 -0.25	0 1 1 1 1 0 0 1	0.625 -0.125 -0.125 0.125 0.125 -0.125 -0.125 -0.375
1 1 1 1 1 0 0 0	0.625 0.125 0.125 0.125 0.375 -0.125 -0.125 -0.125	1 1 1 1 1 0 0 1	0.75 0 0 0.25 0.25 0 0 -0.25
0 0 0 0 0 1 0 0	0.125 -0.125 0.125 -0.125 -0.125 0.125 -0.125 0.125	0 0 0 0 0 1 0 1	0.25 -0.25 0 0 -0.25 0.25 0 0
1 0 0 0 0 1 0 0	0.25 0 0.25 0 0 0.25 0 0.25	1 0 0 0 0 1 0 1	0.375 -0.125 0.125 0.125 -0.125 0.375 0.125 0.125
0 1 0 0 0 1 0 0	0.25 -0.25 0.25 -0.25 0 0 0 0	0 1 0 0 0 1 0 1	0.375 -0.375 0.125 -0.125 -0.125 0.125 0.125 -0.125
1 1 0 0 0 1 0 0	0.375 -0.125 0.375 -0.125 0.125 0.125 0.125 0.125	1 1 0 0 0 1 0 1	0.5 -0.25 0.25 0 0 0.25 0.25 0
0 0 1 0 0 1 0 0	0.25 0 0 -0.25 0 0.25 -0.25 0	0 0 1 0 0 1 0 1	0.375 -0.125 -0.125 -0.125 -0.125 0.375 -0.125 -0.125
1 0 1 0 0 1 0 0	0.375 0.125 0.125 -0.125 0.125 0.375 -0.125 0.125	1 0 1 0 0 1 0 1	0.5 0 0 0 0 0.5 0 0
0 1 1 0 0 1 0 0	0.375 -0.125 0.125 -0.375 0.125 0.125 -0.125 -0.125	0 1 1 0 0 1 0 1	0.5 -0.25 0 -0.25 0 0.25 0 -0.25
1 1 1 0 0 1 0 0	0.5 0 0.25 -0.25 0.25 0.25 0 0	1 1 1 0 0 1 0 1	0.625 -0.125 0.125 -0.125 0.125 0.375 0.125 -0.125
0 0 0 1 0 1 0 0	0.25 -0.25 0 0 0 0 -0.25 0.25	0 0 0 1 0 1 0 1	0.375 -0.375 -0.125 0.125 -0.125 0.125 -0.125 0.125
1 0 0 1 0 1 0 0	0.375 -0.125 0.125 0.125 0.125 0.125 -0.125 0.375	1 0 0 1 0 1 0 1	0.5 -0.25 0 0.25 0 0.25 0 0.25
0 1 0 1 0 1 0 0	0.375 -0.375 0.125 -0.125 0.125 -0.125 -0.125 0.125	0 1 0 1 0 1 0 1	0.5 -0.5 0 0 0 0 0 0
1 1 0 1 0 1 0 0	0.5 -0.25 0.25 0 0.25 0 0 0.25	1 1 0 1 0 1 0 1	0.625 -0.375 0.125 0.125 0.125 0.125 0.125 0.125
0 0 1 1 0 1 0 0	0.375 -0.125 -0.125 -0.125 0.125 0.125 -0.375 0.125	0 0 1 1 0 1 0 1	0.5 -0.25 -0.25 0 0 0.25 -0.25 0
1 0 1 1 0 1 0 0	0.5 0 0 0 0.25 0.25 -0.25 0.25	1 0 1 1 0 1 0 1	0.625 -0.125 -0.125 0.125 0.125 0.375 -0.125 0.125
0 1 1 1 0 1 0 0	0.5 -0.25 0 -0.25 0.25 0 -0.25 0	0 1 1 1 0 1 0 1	0.625 -0.375 -0.125 -0.125 0.125 0.125 -0.125 -0.125
1 1 1 1 0 1 0 0	0.625 -0.125 0.125 -0.125 0.375 0.125 -0.125 0.125	1 1 1 1 0 1 0 1	0.75 -0.25 0 0 0.25 0.25 0 0
0 0 0 0 1 1 0 0	0.25 0 0.25 0 -0.25 0 -0.25 0	0 0 0 0 1 1 0 1	0.375 -0.125 0.125 0.125 -0.375 0.125 -0.125 -0.125
1 0 0 0 1 1 0 0	0.375 0.125 0.375 0.125 -0.125 0.125 -0.125 0.125	1 0 0 0 1 1 0 1	0.5 0 0.25 0.25 -0.25 0.25 0 0
0 1 0 0 1 1 0 0	0.375 -0.125 0.375 -0.125 -0.125 -0.125 -0.125 -0.125	0 1 0 0 1 1 0 1	0.5 -0.25 0.25 0 -0.25 0 0 -0.25
1 1 0 0 1 1 0 0	0.5 0 0.5 0 0 0 0 0	1 1 0 0 1 1 0 1	0.625 -0.125 0.375 0.125 -0.125 0.125 0.125 -0.125
0 0 1 0 1 1 0 0	0.375 0.125 0.125 -0.125 -0.125 0.125 -0.375 -0.125	0 0 1 0 1 1 0 1	0.5 0 0 0 -0.25 0.25 -0.25 -0.25
1 0 1 0 1 1 0 0	0.5 0.25 0.25 0 0 0.25 -0.25 0	1 0 1 0 1 1 0 1	0.625 0.125 0.125 0.125 -0.125 0.375 -0.125 -0.125
0 1 1 0 1 1 0 0	0.5 0 0.25 -0.25 0 0 -0.25 -0.25	0 1 1 0 1 1 0 1	0.625 -0.125 0.125 -0.125 -0.125 0.125 -0.125 -0.375
1 1 1 0 1 1 0 0	0.625 0.125 0.375 -0.125 0.125 0.125 -0.125 -0.125	1 1 1 0 1 1 0 1	0.75 0 0.25 0 0 0.25 0 -0.25
0 0 0 1 1 1 0 0	0.375 -0.125 0.125 0.125 -0.125 -0.125 -0.375 0.125	0 0 0 1 1 1 0 1	0.5 -0.25 0 0.25 -0.25 0 -0.25 0

Continued on next page

Table S3 – continued from previous page

Function	$\alpha_1, \alpha_2, \alpha_3, \alpha_4, \alpha_5, \alpha_6, \alpha_7, \alpha_8$	Function	$\alpha_1, \alpha_2, \alpha_3, \alpha_4, \alpha_5, \alpha_6, \alpha_7, \alpha_8$
1 0 0 1 1 1 0 0	0.5 0 0.25 0.25 0 0 -0.25 0.25	1 0 0 1 1 1 0 1	0.625 -0.125 0.125 0.375 -0.125 0.125 -0.125 0.125
0 1 0 1 1 1 0 0	0.5 -0.25 0.25 0 0 -0.25 -0.25 0	0 1 0 1 1 1 0 1	0.625 -0.375 0.125 0.125 -0.125 -0.125 -0.125 -0.125
1 1 0 1 1 1 0 0	0.625 -0.125 0.375 0.125 0.125 -0.125 -0.125 0.125	1 1 0 1 1 1 0 1	0.75 -0.25 0.25 0.25 0 0 0 0
0 0 1 1 1 1 0 0	0.5 0 0 0 0 0 -0.5 0	0 0 1 1 1 1 0 1	0.625 -0.125 -0.125 0.125 -0.125 0.125 -0.375 -0.125
1 0 1 1 1 1 0 0	0.625 0.125 0.125 0.125 0.125 0.125 -0.375 0.125	1 0 1 1 1 1 0 1	0.75 0 0 0.25 0 0.25 -0.25 0
0 1 1 1 1 1 0 0	0.625 -0.125 0.125 -0.125 0.125 -0.125 -0.375 -0.125	0 1 1 1 1 1 0 1	0.75 -0.25 0 0 0 0 -0.25 -0.25
1 1 1 1 1 1 0 0	0.75 0 0.25 0 0.25 0 -0.25 0	1 1 1 1 1 1 0 1	0.875 -0.125 0.125 0.125 0.125 0.125 -0.125 -0.125
0 0 0 0 0 0 1 0	0.125 0.125 -0.125 -0.125 -0.125 -0.125 0.125 0.125	0 0 0 0 0 0 1 1	0.25 0 -0.25 0 -0.25 0 0.25 0
1 0 0 0 0 0 1 0	0.25 0.25 0 0 0 0 0.25 0.25	1 0 0 0 0 0 1 1	0.375 0.125 -0.125 0.125 -0.125 0.125 0.375 0.125
0 1 0 0 0 0 1 0	0.25 0 0 -0.25 0 -0.25 0.25 0	0 1 0 0 0 0 1 1	0.375 -0.125 -0.125 -0.125 -0.125 -0.125 0.375 -0.125
1 1 0 0 0 0 1 0	0.375 0.125 0.125 -0.125 0.125 -0.125 0.375 0.125	1 1 0 0 0 0 1 1	0.5 0 0 0 0 0 0.5 0
0 0 1 0 0 0 1 0	0.25 0.25 -0.25 -0.25 0 0 0 0	0 0 1 0 0 0 1 1	0.375 0.125 -0.375 -0.125 -0.125 0.125 0.125 -0.125
1 0 1 0 0 0 1 0	0.375 0.375 -0.125 -0.125 0.125 0.125 0.125 0.125	1 0 1 0 0 0 1 1	0.5 0.25 -0.25 0 0 0.25 0.25 0
0 1 1 0 0 0 1 0	0.375 0.125 -0.125 -0.375 0.125 -0.125 0.125 -0.125	0 1 1 0 0 0 1 1	0.5 0 -0.25 -0.25 0 0 0.25 -0.25
1 1 1 0 0 0 1 0	0.5 0.25 0 -0.25 0.25 0 0.25 0	1 1 1 0 0 0 1 1	0.625 0.125 -0.125 -0.125 0.125 0.125 0.375 -0.125
0 0 0 1 0 0 1 0	0.25 0 -0.25 0 0 -0.25 0 0.25	0 0 0 1 0 0 1 1	0.375 -0.125 -0.375 0.125 -0.125 -0.125 0.125 0.125
1 0 0 1 0 0 1 0	0.375 0.125 -0.125 0.125 0.125 -0.125 0.125 0.375	1 0 0 1 0 0 1 1	0.5 0 -0.25 0.25 0 0 0.25 0.25
0 1 0 1 0 0 1 0	0.375 -0.125 -0.125 -0.125 0.125 -0.375 0.125 0.125	0 1 0 1 0 0 1 1	0.5 -0.25 -0.25 0 0 -0.25 0.25 0
1 1 0 1 0 0 1 0	0.5 0 0 0 0.25 -0.25 0.25 0.25	1 1 0 1 0 0 1 1	0.625 -0.125 -0.125 0.125 0.125 -0.125 0.375 0.125
0 0 1 1 0 0 1 0	0.375 0.125 -0.375 -0.125 0.125 -0.125 -0.125 0.125	0 0 1 1 0 0 1 1	0.5 0 -0.5 0 0 0 0 0
1 0 1 1 0 0 1 0	0.5 0.25 -0.25 0 0.25 0 0 0.25	1 0 1 1 0 0 1 1	0.625 0.125 -0.375 0.125 0.125 0.125 0.125 0.125
0 1 1 1 0 0 1 0	0.5 0 -0.25 -0.25 0.25 -0.25 0 0	0 1 1 1 0 0 1 1	0.625 -0.125 -0.375 -0.125 0.125 -0.125 0.125 -0.125
1 1 1 1 0 0 1 0	0.625 0.125 -0.125 -0.125 0.375 -0.125 0.125 0.125	1 1 1 1 0 0 1 1	0.75 0 -0.25 0 0.25 0 0.25 0
0 0 0 0 1 0 1 0	0.25 0.25 0 0 -0.25 -0.25 0 0	0 0 0 0 1 0 1 1	0.375 0.125 -0.125 0.125 -0.375 -0.125 0.125 -0.125
1 0 0 0 1 0 1 0	0.375 0.375 0.125 0.125 -0.125 -0.125 0.125 0.125	1 0 0 0 1 0 1 1	0.5 0.25 0 0.25 -0.25 0 0.25 0
0 1 0 0 1 0 1 0	0.375 0.125 0.125 -0.125 -0.125 -0.375 0.125 -0.125	0 1 0 0 1 0 1 1	0.5 0 0 0 -0.25 -0.25 0.25 -0.25
1 1 0 0 1 0 1 0	0.5 0.25 0.25 0 0 -0.25 0.25 0	1 1 0 0 1 0 1 1	0.625 0.125 0.125 0.125 -0.125 -0.125 0.375 -0.125
0 0 1 0 1 0 1 0	0.375 0.375 -0.125 -0.125 -0.125 -0.125 -0.125 -0.125	0 0 1 0 1 0 1 1	0.5 0.25 -0.25 0 -0.25 0 0 -0.25
1 0 1 0 1 0 1 0	0.5 0.5 0 0 0 0 0 0	1 0 1 0 1 0 1 1	0.625 0.375 -0.125 0.125 -0.125 0.125 0.125 -0.125
0 1 1 0 1 0 1 0	0.5 0.25 0 -0.25 0 -0.25 0 -0.25	0 1 1 0 1 0 1 1	0.625 0.125 -0.125 -0.125 -0.125 -0.125 0.125 -0.375

Continued on next page

Table S3 – continued from previous page

Function	$\alpha_1, \alpha_2, \alpha_3, \alpha_4, \alpha_5, \alpha_6, \alpha_7, \alpha_8$	Function	$\alpha_1, \alpha_2, \alpha_3, \alpha_4, \alpha_5, \alpha_6, \alpha_7, \alpha_8$
1 1 1 0 1 0 1 0	0.625 0.375 0.125 -0.125 0.125 -0.125 0.125 -0.125	1 1 1 0 1 0 1 1	0.75 0.25 0 0 0 0 0.25 -0.25
0 0 0 1 1 0 1 0	0.375 0.125 -0.125 0.125 -0.125 -0.375 -0.125 0.125	0 0 0 1 1 0 1 1	0.5 0 -0.25 0.25 -0.25 -0.25 0 0
1 0 0 1 1 0 1 0	0.5 0.25 0 0.25 0 -0.25 0 0.25	1 0 0 1 1 0 1 1	0.625 0.125 -0.125 0.375 -0.125 -0.125 0.125 0.125
0 1 0 1 1 0 1 0	0.5 0 0 0 0 -0.5 0 0	0 1 0 1 1 0 1 1	0.625 -0.125 -0.125 0.125 -0.125 -0.375 0.125 -0.125
1 1 0 1 1 0 1 0	0.625 0.125 0.125 0.125 0.125 -0.375 0.125 0.125	1 1 0 1 1 0 1 1	0.75 0 0 0.25 0 -0.25 0.25 0
0 0 1 1 1 0 1 0	0.5 0.25 -0.25 0 0 -0.25 -0.25 0	0 0 1 1 1 0 1 1	0.625 0.125 -0.375 0.125 -0.125 -0.125 -0.125 -0.125
1 0 1 1 1 0 1 0	0.625 0.375 -0.125 0.125 0.125 -0.125 -0.125 0.125	1 0 1 1 1 0 1 1	0.75 0.25 -0.25 0.25 0 0 0 0
0 1 1 1 1 0 1 0	0.625 0.125 -0.125 -0.125 0.125 -0.375 -0.125 -0.125	0 1 1 1 1 0 1 1	0.75 0 -0.25 0 0 -0.25 0 -0.25
1 1 1 1 1 0 1 0	0.75 0.25 0 0 0.25 -0.25 0 0	1 1 1 1 1 0 1 1	0.875 0.125 -0.125 0.125 0.125 -0.125 0.125 -0.125
0 0 0 0 0 1 1 0	0.25 0 0 -0.25 -0.25 0 0 0.25	0 0 0 0 0 1 1 1	0.375 -0.125 -0.125 -0.125 -0.375 0.125 0.125 0.125
1 0 0 0 0 1 1 0	0.375 0.125 0.125 -0.125 -0.125 0.125 0.125 0.375	1 0 0 0 0 1 1 1	0.5 0 0 0 -0.25 0.25 0.25 0.25
0 1 0 0 0 1 1 0	0.375 -0.125 0.125 -0.375 -0.125 -0.125 0.125 0.125	0 1 0 0 0 1 1 1	0.5 -0.25 0 -0.25 -0.25 0 0.25 0
1 1 0 0 0 1 1 0	0.5 0 0.25 -0.25 0 0 0.25 0.25	1 1 0 0 0 1 1 1	0.625 -0.125 0.125 -0.125 -0.125 0.125 0.375 0.125
0 0 1 0 0 1 1 0	0.375 0.125 -0.125 -0.375 -0.125 0.125 -0.125 0.125	0 0 1 0 0 1 1 1	0.5 0 -0.25 -0.25 -0.25 0.25 0 0
1 0 1 0 0 1 1 0	0.5 0.25 0 -0.25 0 0.25 0 0.25	1 0 1 0 0 1 1 1	0.625 0.125 -0.125 -0.125 -0.125 0.375 0.125 0.125
0 1 1 0 0 1 1 0	0.5 0 0 -0.5 0 0 0 0	0 1 1 0 0 1 1 1	0.625 -0.125 -0.125 -0.375 -0.125 0.125 0.125 -0.125
1 1 1 0 0 1 1 0	0.625 0.125 0.125 -0.375 0.125 0.125 0.125 0.125	1 1 1 0 0 1 1 1	0.75 0 0 -0.25 0 0.25 0.25 0
0 0 0 1 0 1 1 0	0.375 -0.125 -0.125 -0.125 -0.125 -0.125 -0.125 0.375	0 0 0 1 0 1 1 1	0.5 -0.25 -0.25 0 -0.25 0 0 0.25
1 0 0 1 0 1 1 0	0.5 0 0 0 0 0 0 0.5	1 0 0 1 0 1 1 1	0.625 -0.125 -0.125 0.125 -0.125 0.125 0.125 0.375
0 1 0 1 0 1 1 0	0.5 -0.25 0 -0.25 0 -0.25 0 0.25	0 1 0 1 0 1 1 1	0.625 -0.375 -0.125 -0.125 -0.125 -0.125 0.125 0.125
1 1 0 1 0 1 1 0	0.625 -0.125 0.125 -0.125 0.125 -0.125 0.125 0.375	1 1 0 1 0 1 1 1	0.75 -0.25 0 0 0 0 0.25 0.25
0 0 1 1 0 1 1 0	0.5 0 -0.25 -0.25 0 0 -0.25 0.25	0 0 1 1 0 1 1 1	0.625 -0.125 -0.375 -0.125 -0.125 0.125 -0.125 0.125
1 0 1 1 0 1 1 0	0.625 0.125 -0.125 -0.125 0.125 0.125 -0.125 0.375	1 0 1 1 0 1 1 1	0.75 0 -0.25 0 0 0.25 0 0.25
0 1 1 1 0 1 1 0	0.625 -0.125 -0.125 -0.375 0.125 -0.125 -0.125 0.125	0 1 1 1 0 1 1 1	0.75 -0.25 -0.25 -0.25 0 0 0 0
1 1 1 1 0 1 1 0	0.75 0 0 -0.25 0.25 0 0 0.25	1 1 1 1 0 1 1 1	0.875 -0.125 -0.125 -0.125 0.125 0.125 0.125 0.125
0 0 0 0 1 1 1 0	0.375 0.125 0.125 -0.125 -0.375 -0.125 -0.125 0.125	0 0 0 0 1 1 1 1	0.5 0 0 0 -0.5 0 0 0
1 0 0 0 1 1 1 0	0.5 0.25 0.25 0 -0.25 0 0 0.25	1 0 0 0 1 1 1 1	0.625 0.125 0.125 0.125 -0.375 0.125 0.125 0.125
0 1 0 0 1 1 1 0	0.5 0 0.25 -0.25 -0.25 -0.25 0 0	0 1 0 0 1 1 1 1	0.625 -0.125 0.125 -0.125 -0.375 -0.125 0.125 -0.125
1 1 0 0 1 1 1 0	0.625 0.125 0.375 -0.125 -0.125 -0.125 0.125 0.125	1 1 0 0 1 1 1 1	0.75 0 0.25 0 -0.25 0 0.25 0
0 0 1 0 1 1 1 0	0.5 0.25 0 -0.25 -0.25 0 -0.25 0	0 0 1 0 1 1 1 1	0.625 0.125 -0.125 -0.125 -0.375 0.125 -0.125 -0.125

Continued on next page

Table S3 – continued from previous page

Function	$\alpha_1, \alpha_2, \alpha_3, \alpha_4, \alpha_5, \alpha_6, \alpha_7, \alpha_8$	Function	$\alpha_1, \alpha_2, \alpha_3, \alpha_4, \alpha_5, \alpha_6, \alpha_7, \alpha_8$
1 0 1 0 1 1 1 0	0.625 0.375 0.125 -0.125 -0.125 0.125 -0.125 0.125	1 0 1 0 1 1 1 1	0.75 0.25 0 0 -0.25 0.25 0 0
0 1 1 0 1 1 1 0	0.625 0.125 0.125 -0.375 -0.125 -0.125 -0.125 -0.125	0 1 1 0 1 1 1 1	0.75 0 0 -0.25 -0.25 0 0 -0.25
1 1 1 0 1 1 1 0	0.75 0.25 0.25 -0.25 0 0 0 0	1 1 1 0 1 1 1 1	0.875 0.125 0.125 -0.125 -0.125 0.125 0.125 -0.125
0 0 0 1 1 1 1 0	0.5 0 0 0 -0.25 -0.25 -0.25 0.25	0 0 0 1 1 1 1 1	0.625 -0.125 -0.125 0.125 -0.375 -0.125 -0.125 0.125
1 0 0 1 1 1 1 0	0.625 0.125 0.125 0.125 -0.125 -0.125 -0.125 0.375	1 0 0 1 1 1 1 1	0.75 0 0 0.25 -0.25 0 0 0.25
0 1 0 1 1 1 1 0	0.625 -0.125 0.125 -0.125 -0.125 -0.375 -0.125 0.125	0 1 0 1 1 1 1 1	0.75 -0.25 0 0 -0.25 -0.25 0 0
1 1 0 1 1 1 1 0	0.75 0 0.25 0 0 -0.25 0 0.25	1 1 0 1 1 1 1 1	0.875 -0.125 0.125 0.125 -0.125 -0.125 0.125 0.125
0 0 1 1 1 1 1 0	0.625 0.125 -0.125 -0.125 -0.125 -0.125 -0.375 0.125	0 0 1 1 1 1 1 1	0.75 0 -0.25 0 -0.25 0 -0.25 0
1 0 1 1 1 1 1 0	0.75 0.25 0 0 0 0 -0.25 0.25	1 0 1 1 1 1 1 1	0.875 0.125 -0.125 0.125 -0.125 0.125 -0.125 0.125
0 1 1 1 1 1 1 0	0.75 0 0 -0.25 0 -0.25 -0.25 0	0 1 1 1 1 1 1 1	0.875 -0.125 -0.125 -0.125 -0.125 -0.125 -0.125 -0.125
1 1 1 1 1 1 1 0	0.875 0.125 0.125 -0.125 0.125 -0.125 -0.125 0.125	1 1 1 1 1 1 1 1	1 0 0 0 0 0 0 0

In Appendix-2 we train multiple times the GN to learn all the binary and 3-ary boolean functions using SGD. Our experimental results show that for the 3-ary boolean function space,the GN could learn at most 114 the 256 functions. This results provides an empirical proof of our assumption that a single unit of GN is not expressive enough to learn any k-ary boolean funciton.

2 APPENDIX 2

Results of training the Generalized Neuron (GN) when trained to learn all functions of the binary and ternary function space. The training method used in this section was back-propagation using an Adam optimizer with no drop-out regularization and a fixed learning rate of .1. All algorithms were trained using the mean squared error loss function, and all the results in this appendix are the best-obtained results from running the experiments 5 consecutive times.

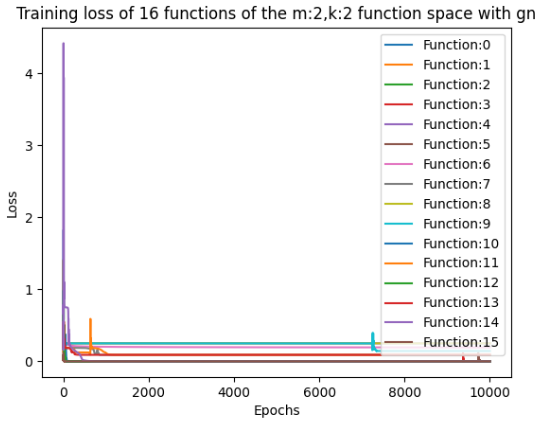

Figure S1. Figure that plots the loss function of the GN when learning the complete binary Boolean space using ADAM optimizer for a total of 10000 episodes and learning rate of 0.1

Table S4. Average final loss and amount of learned functions for the binary Boolean function space using the Generalized neuron. The average and number of learned functions are taken from the highest obtained after running the experiments for 5 consecutive times.

Epochs	Average Loss	Number of learned functions
100	0.0213	11
1000	0.0661	13
10000	0.0792	14

Table S5. Average final loss and amount of learned functions for the ternary Boolean function space using the Generalized neuron. The average and number of learned functions are taken from the highest obtained after running the experiments for 5 consecutive times.

Epochs	Average Loss	Number of learned functions
100	0.0907	60
1000	0.0811	103
2000	0.0798	114

REFERENCES

Srivastava, N., Hinton, G., Krizhevsky, A., Sutskever, I., and Salakhutdinov, R. (2014). Dropout: A simple way to prevent neural networks from overfitting. *J. Mach. Learn. Res.* 15, 1929–1958. doi:10.5555/2627435.2670313
