# Supplementary figures and images for: Signal Perceptron: On the Identifiability of Boolean Function Spaces and Beyond
Source: Front Artif Intell. 2022 Jun 2;5:770254. doi: 10.3389/frai.2022.770254 (PMC9203047; doi:10.3389/frai.2022.770254)

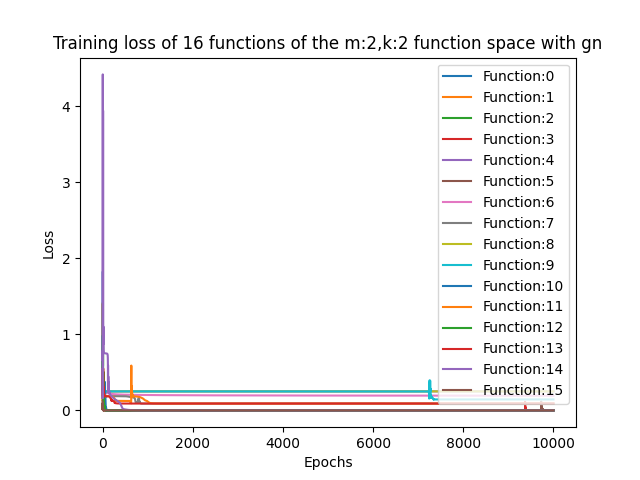

Supplement: Supplementary file 1 [file Data_Sheet_1.zip › SignalPerceptron_SupplementaryMatterial/gn.png]

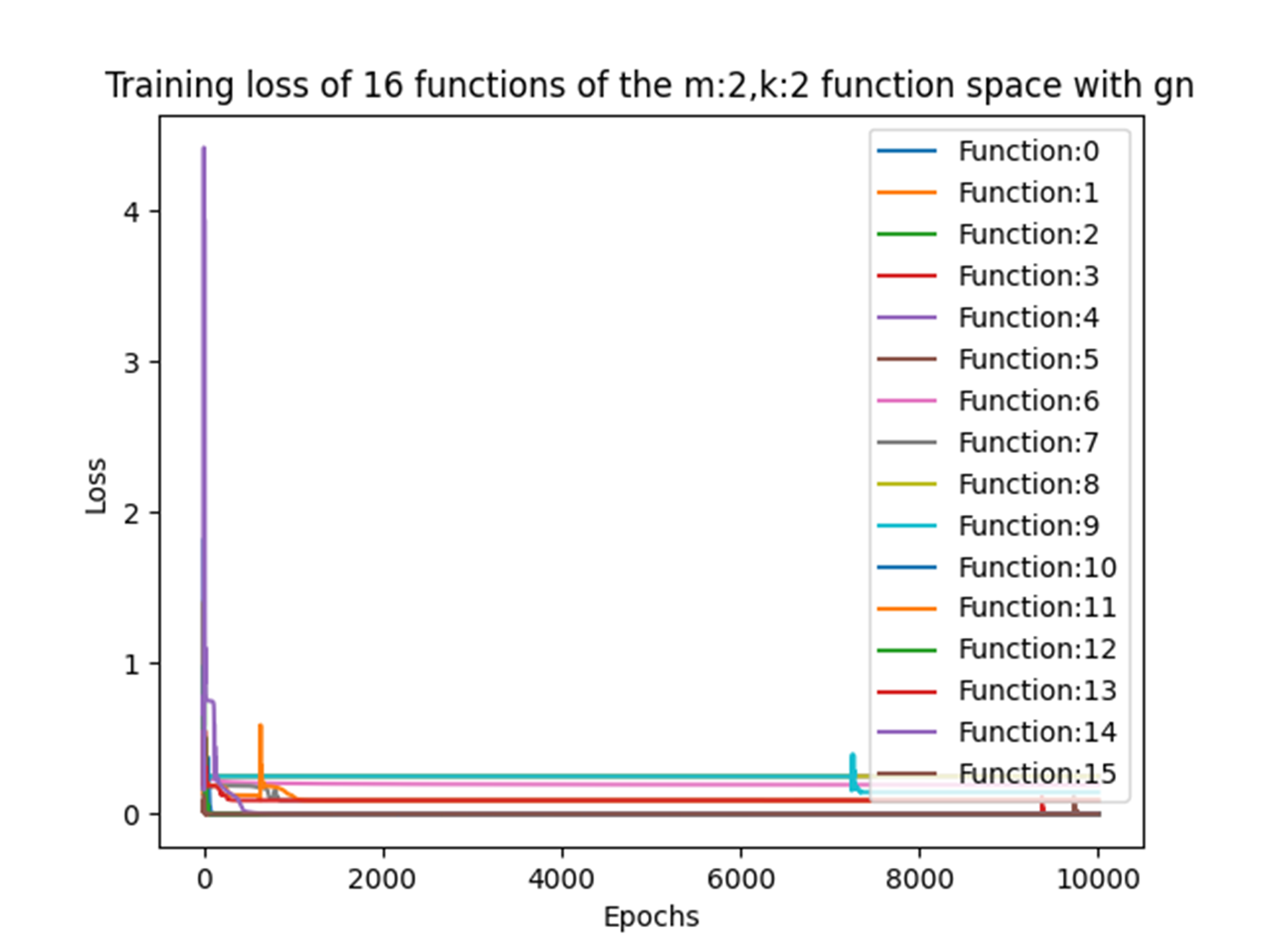

Supplement: Supplementary file 2 [file Image_1.png]
